# Association of blood pressure and long‐term change with chronic kidney disease risk among Chinese adults with different glucose metabolism according to the 2017 ACC/AHA guidelines

**DOI:** 10.1111/jch.14371

**Published:** 2021-11-12

**Authors:** Jia He, Zhaoyang Li, Ruixin Wang, Hongli Nie, Fei Wang, Jing Yuan, Xiaoping Miao, Ping Yao, Sheng Wei, Xiaomin Zhang, Huan Guo, Handong Yang, Tangchun Wu, Meian He

**Affiliations:** ^1^ Department of Occupational and Environmental Health and State Key Laboratory of Environmental Health for Incubating School of Public Health Tongji Medical College Huazhong University of Science and Technology Wuhan Hubei China; ^2^ Dongfeng Central Hospital Dongfeng Motor Corporation and Hubei University of Medicine Shiyan Hubei China

**Keywords:** 2017 ACC/AHA hypertension guidelines, blood pressure, chronic kidney disease, glucose metabolism, long‐term blood pressure change

## Abstract

Whether the definition of hypertension according to 2017 AHA/ACC guidelines and blood pressure (BP) changes was related to the increased risk of chronic kidney disease (CKD) remained debated. This prospective cohort study aimed to investigate the association of BP and long‐term BP change with CKD risk with different glucose metabolism according to the new hypertension guidelines. This study examined 12 951 participants and 11 183 participants derived from the older people cohort study, respectively. Participants were divided into three groups based on blood glucose and the risks were assessmented by the logistic regression model. During a 10 years of follow‐up period, 2727 individuals developed CKD (21.1%). Compared with those with BP < 130/80 mmHg, individuals with increased BP levels had significantly increased risk of incident CKD. Participants with BP of 130–139/80–89 or ≥140/90 mmHg had 1.51‐ and 1.89‐fold incident risk of CKD in patients with diabetes mellitus (DM). Compared with individuals with stable BP (−5 to 5 mmHg), the risk of CKD was reduced when BP decreased by 5 mmHg or more and increased when BP increased ≥5 mmHg among normoglycemia and prediabetes participants. Similar results were observed for rapid estimated glomerular filtration rate (eGFR) decline. In conclusion, the BP of 130–139/80–89 mmHg combined with prediabetes or DM had an increased risk of incident CKD and rapid eGFR decline in older people. Long‐term changes of BP by more than 5 mmHg among normoglycemia or prediabetes were associated with the risk of incident CKD and rapid eGFR decline.

## 1 INTRODUCTION

Chronic kidney disease (CKD) is an important global public health problem. It affects 10–15% of the population worldwide and is now recognized as the most rapidly increasing contributor to global burden of disease.^1^, ^2^ Diabetes mellitus (DM) and hypertension are two of the most powerful risk factors of CKD.^3^ From a public health perspective, effective and safe control of blood pressure (BP) and blood glucose is essential for the prevention and treatment of CKD.

In 2017, the American College of Cardiology and the American Heart Association (ACC/AHA) Guideline on BP in adults was released^4^; however, there has been controversy surrounding the applicability of the guidelines' definition of hypertension (BP ≥ 130/80 mmHg).^5^, ^6^, ^7^, ^8^, ^9^ Whether the new definition of hypertension was related to the increased risk of CKD remained debated,^10^, ^11^, ^12^, ^13^ and data are lacking on what extent these BP stratums affect CKD risk in Chinese adults. More importantly, there has been disagreement with BP reduction for CKD risk with different glycemic status.^14^, ^15^ It was showed that intensive lowering of BP (target BP less than 120/80 mmHg) did not provide significant benefit for CKD development among patients with both CKD and hypertension but not with diabetes.^16^ However, a recent study reported intensive lowering of BP increased incident CKD risk in individuals with and without type 2 diabetes.^17^ Meanwhile, the impact of prediabetes on the effects of BP control on kidney events also remained uncertain.^18^ Therefore, it is necessary to investigate how BP levels of 130–139/80–89 mmHg affect CKD risks with different glycemic status.

In addition, BP levels at a single time point may not be sufficient to evaluate the disease risk. Prospective studies have pointed out that a worse change in BP control categories over 1 year was associated with increased occurrence of renal outcomes.^19^ However, different initial BP and observation time will affect the effect of BP changes on CKD.^20^ Meanwhile, there is limited evidence on the relationships between long‐term BP changes on CKD in individuals with different glycemic status, especially when BP is elevated to the hypertension category defined by the 2017 ACC/AHA.

In the current study, we aimed to explore the association of newly defined hypertension criteria by the 2017 ACC/AHA and long‐term BP changes with 10‐year incident CKD risk in middle‐aged and elderly Chinese with different glycemic status.

## 2 METHODS

### 2.1 Data source and study population

All participants of this study were derived from the Dongfeng‐Tongji (DFTJ) cohort and the detailed baseline profiles have been described previously.^21^ This study followed the Strengthening the Reporting of Observational Studies in Epidemiology (STROBE) reporting guideline. Briefly, a total of 27 009 retired employees were recruited in the cohort and completed baseline questionnaires, medical examinations, and provided baseline blood samples between September 2008 and June 2010. The participants were invited to the follow‐up survey every 5 years (2013 and 2018) and trained investigators collected epidemiological data via questionnaires by face‐to‐face interviews. Among 27 009 individuals who with CKD (*n* = 2911), cancer (*n* = 899), and self‐reported kidney disease (*n* = 988) at baseline, as well as those with missing values of serum creatinine (*n* = 7845), BP (*n* = 536), and other covariates (*n* = 2402) were excluded, resulting in a final study sample of 12 951 participants for analysis of 10‐year incident CKD risk (Figure S1).

To explore the association of long‐term BP changes with risk of incident CKD, individuals who participated in both baseline and first follow‐up surveys (*n* = 24 601) were included. After excluding individuals with CKD (*n* = 5564), cancer (*n* = 1992), and self‐reported kidney disease (*n* = 1629) at or prior to the follow‐up in 2013, and missing data of serum creatinine (*n* = 5999), BP (*n* = 428), and other covariates (*n* = 1937), finally 11 183 participants were remained for the further analysis (Figure S2).

The present study has been approved by the Ethics and Human Subject Committee of the School of Public Health, Tongji Medical College, Huazhong University of Science and Technology, and Dongfeng General Hospital, the Dongfeng Motor Corporation (DMC). All study participants provided written informed consents.

### 2.2 BP and blood glucose measurement

BP was measured at the right arm after a 5 min rest period of sitting position via a mercury sphygmomanometer (Shanghai Zhangdong Med‐Tech Ltd Company, China) by trained nurses or physicians and the cuff size was selected based on the circumference of the upper arm. BP levels were measured at baseline and follow‐ups and long‐term BP change levels were calculated as BP in 2013 − BP in 2008. According to the AHA/ACC guidelines,^4^ BP was classified as normal or elevated (<130/80 mmHg), stage 1 hypertension (130–139/80–89 mmHg), or stage 2 hypertension (≥140/90 mmHg or treated). Blood samples were collected after an overnight fast and fasting plasma glucose (FPG) were determined by Glucose Oxidase method by Abbott Aroset analyzer (Abbott Laboratories, Abbott Park, IL, USA) at hospital's laboratory. DM was defined by FPG level ≥ 7.0 mmol/L, self‐reported physician diagnosis of diabetes, or use of antidiabetic medication at baseline. Prediabetes was defined as FPG between 5.6 and 7.0 mmol/L. Normoglycemia was defined by FPG < 5.6 mmol/L.^22^

### 2.3 Follow‐up and outcome measurement

The outcome of the present analysis was incident CKD between baseline 2008 and December 31, 2018. Incident CKD was judged through the estimated glomerular filtration rate (eGFR) calculated with the formula of Chronic Kidney Disease Epidemiology Collaboration, and defined as the occurrence of eGFR < 60 ml/min/1.73 m^2^ in the follow‐ups.^23^ Furthermore, the death for CKD were confirmed by DMC's health care service system, medical record reviews in the DMC‐owned hospitals, and death certificates up to December 31, 2018 and were adjudicated by an expert panel of physicians according to International Classification of Diseases, Tenth Revision (ICD‐10) codes (D63.1, E10.2, E11.2, E12.2, E13.2, E14.2, I12‐I13.9, N02‐N08.8, N15.0, N18‐N18.9, Q61‐Q62.8).^24^

### 2.4 Covariates

The epidemiological questionnaires were used to collect information on demographic characteristics (age, sex, marital status, and education level), lifestyle (smoking status, drinking status, and physical activity), and family and personal disease histories by trained interviewers. Current smokers were defined as participants who were smoking at least one cigarette per day for more than half a year. Those who drink at least one time per week for more than half a year were considered as current drinkers. Physical activity was ascertained by regular exercise for at least 20 min per time over the past 6 months.

Physical examinations (standing height, body weight, systolic blood pressure [SBP], and diastolic blood pressure [DBP]) were performed by trained physicians and nurses. Levels of triglycerides (TG), total cholesterol (TC), and serum creatinine were measured at the hospital's laboratory following standard laboratory procedures. Hyperlipidemia was defined as TG > 1.70 mmol/L or TC > 5.72 mmol/L, previous physician diagnosis of hyperlipidemia, or current using lipid‐lowering medication.^25^

### 2.5 Statistical analysis

All statistical analysis was performed from February 1 to October 30, 2020 using SPSS software (version 23.0, IBM‐SPSS, Chicago, IL, USA) and R software (version 3.6.1, R Foundation for Statistical Computing). Comparison of the differences of demographic characteristics across BP categories were conducted using analysis of variance for continuous variables (presented in mean ± SD) and chi‐squared analysis for categorical variables (presented in number [percentages]).

Relative risks (RRs) and 95% confidence intervals (95% CIs) of incident CKD were estimated using logistic regression. Model 1 adjusted for demographic characteristics including age and sex. Model 2 additionally adjusted for body mass index (BMI), TC, and TG. Based on model 2, model 3 additionally adjusted for education degree, antidiabetic medications, antihypertensive medications, lifestyle behaviors including physical activity, smoking, and drinking status.

Restricted cubic spline regression (RCS) linked to logistic regression models were conducted to accommodate nonlinearity in the associations of continuous BP values and CKD incidence.

To evaluate the association of long‐term BP change with CKD at different blood glucose categories, we constructed three categories of SBP or DBP changes as <−5, −5 to 5, and ≥5 mmHg. Multivariate logistic regression models were performed to calculate RRs (95% CIs) after adjusted for covariates (BP change of −5 to 5 mmHg as reference). In addition, stratified analyses were conducted by sex (men, women), age (<60 years, ≥60 years), BMI (<24 kg/m^2^, ≥24 kg/m^2^), smoking status (never‐smokers, ever‐smokers), drinking status (never‐drinkers, ever‐drinkers), baseline renal function (eGFR < 90 ml/min/1.73 m^2^, eGFR ≥ 90 ml/min/1.73 m^2^). Potential interactions were tested by adding the interaction product of each subgroup with BP categories and changes into the model.

In the sensitivity analyses, we repeated the multivariable logistic regression analyses to test the 5‐year risks of CKD (2008–2013) and the 10‐year risks of CKD (2008–2018) through divide BP levels into <120/80 (reference group), 120–129/<80, 130–139/80–89, 140–159/90–99, and ≥160/100 mmHg groups, construct categories of SBP or DBP changes as <−15, −15 to −5, −5 to 5 (reference group), 5–15, and ≥15 mmHg, and analyze in those without use of antihypertensive or antidiabetic medications at baseline. We also investigated the RRs (95% CIs) of the rapid eGFR decline (average eGFR reduction > 3 ml/min/1.73 m^2^/year)^26^ according to different glycemic categories and BP (categories and changes). All *p*‐values were two‐sided, and *p* < .05 was considered statistically significant.

## 3 RESULTS

Distribution of the characteristics by BP categories at baseline is presented in Table 1. Among 12 951 participants (mean [SD] age, 61.8 [6.9] years; 5523 [42.6%] men), the proportion of stage 1 hypertension and stage 2 hypertension according to the new 2017 ACC/AHA hypertension guidelines were 29.9% and 34.1%, respectively. Participants with higher BP levels were more likely to be older, men, lower education, current drinkers, with higher levels of BMI, FPG, TC, and TG, lower levels of eGFR, and have higher proportion of prediabetes, diabetes, and hyperlipidemia (Table 1).

**TABLE 1:** Basic characteristics according to blood pressure categories

		SBP/DBP categories (mmHg)			
Variables	Overall	<130/80	130–139/80–89	≥140/90	*p‐*value
Number of participants	12 951	4667	3873	4411	
Age (years)	61.8 ± 6.9	60.9 ± 7.2	61.7 ± 6.8	62.8 ± 6.5	<.001
Men [*n* (%)]	5523(42.6)	1858(39.8)	1630(42.1)	2035(46.1)	<.001
Education [*n* (%)]					<.001
Primary school	3644(28.1)	1205(25.8)	1042(26.9)	1397(31.7)	
Middle school	4849(37.4)	1642(35.2)	1505(38.9)	1702(38.6)	
High school or above	4458(34.4)	1820(39.0)	1326(34.2)	1312(29.7)	
Physical activity [*n* (%)]	11 645(89.9)	4211(90.2)	3488(90.1)	3946(89.5)	.447
Current smoker [*n* (%)]	2185(16.9)	861(18.4)	645(16.7)	679(15.4)	<.001
Current drinker [*n* (%)]	2818(21.8)	969(20.8)	808(20.9)	1041(23.6)	<.001
BMI (kg/m2)	24.5 ± 3.3	23.6 ± 3.1	24.7 ± 3.2	25.2 ± 3.3	<.001
SBP (mmHg)	127.9 ± 18	112.0 ± 9.1	125.5 ± 7.6	147.0 ± 13.4	<.001
DBP (mmHg)	77.4 ± 10.7	68.1 ± 5.1	78.0 ± 5.4	86.7 ± 10.2	<.001
FPG (mmol/L)	6.0 ± 1.5	5.8 ± 1.3	5.9 ± 1.5	6.2 ± 1.7	<.001
eGFR (ml/min/1.73 m^2^)	83.5 ± 17.9	84.1 ± 12.6	83.3 ± 12.3	82.9 ± 25.2	.003
TC (mmol/L)	5.2 ± 1.0	5.1 ± 0.9	5.2 ± 1.0	5.2 ± 1.0	<.001
TG (mmol/L)	1.4 ± 1.0	1.3 ± 0.8	1.4 ± 0.9	1.6 ± 1.2	<.001
Glucose status					<.001
Normoglycemia [*n* (%)]	5896(45.5)	2368(50.7)	1780(46.0)	1748(39.6)	
Prediabetes [*n* (%)]	4868(37.6)	1667(35.7)	1451(37.5)	1750(39.7)	
Diabetes [*n* (%)]	2187(16.9)	632(13.5)	642(16.6)	913(20.7)	
Hyperlipidemia [*n* (%)]	6611(51.0)	2030(43.5)	2067(53.4)	2514(57.0)	<.001
Antidiabetic medications [*n* (%)]	1033(8.0)	301(6.4)	309(8.0)	423(9.6)	<.001
Antihypertensive medications [*n* (%)]	3471(26.8)	612(13.1)	1022(26.4)	1837(41.6)	<.001

Abbreviations: BMI, body mass index; DBP, diastolic blood pressure; eGFR, estimated glomerular filtration rate; FPG, fasting plasma glucose; SBP, systolic blood pressure; TC, total cholesterol; TG, triglyceride.

During a 10 years of follow‐up period, 2727 individuals developed CKD (21.1%). Among the incident CKD cases, 1272 (46.6%) were normoglycemia at baseline, 920 (33.7%) participants with prediabetes, and 535 (19.6%) with DM. Among the total participants, compared with those with BP < 130/80 mmHg, individuals with increased BP levels had significantly increased risk of incident CKD. The RRs (95% CIs) of CKD in individuals with BP of 130–139/80–89 or ≥140/90 mmHg were 1.17 (1.04–1.31) and 1.59 (1.42–1.77), respectively. Similar findings were obtained in different glycemic groups (all *p*_trend_ < .001). In those with DM, participants with BP of 130–139/80–89 or ≥140/90 mmHg had 1.51‐ and 1.89‐fold incident risk of CKD (Table 2). Similar results were observed when we evaluated the association during a 5‐year follow‐up period (2008–2013) (Table S1). After exclusion of participants who were taking antihypertensive or antidiabetic medications at baseline, the significant associations still remained in both 10‐year and 5‐year follow‐up periods (Tables S2–S3). We further divided BP levels into five groups, incident risk of CKD increased with BP levels increased (all *p*_trend_ < .001) among the whole participants, participants with normoglycemia, prediabetes, and diabetes groups (Tables S4–S5).

**TABLE 2:** Multivariable‐adjusted RR of incident CKD risk according to different glycemic categories and BP categories (2008–2018)^a^

				Adjusted RR (95% CI)		
BP category (mmHg)	No./total	Rate	Crude RR (95% CI)	Model 1^b^	Model 2^c^	Model 3^d^
Total	2727/12 951	21.1				
<130/80	750/4667	16.1	1.00 (Reference)	1.00 (Reference)	1.00 (Reference)	1.00 (Reference)
130–139/80–89	776/3873	20.0	1.31(1.17–1.46)	1.28(1.14–1.43)	1.20(1.07–1.35)	1.17(1.04–1.31)
≥140/90	1201/4411	27.2	1.95(1.76–2.17)	1.85(1.66–2.05)	1.67(1.50–1.86)	1.59(1.42–1.77)
*p*_trend_		<.001	<.001	<.001	<.001	<.001
Normoglycemia	1272/5896	21.6				
<130/80	409/2368	17.3	1.00 (Reference)	1.00 (Reference)	1.00 (Reference)	1.00 (Reference)
130–139/80–89	358/1780	20.1	1.21(1.03–1.41)	1.18(1.01–1.38)	1.11(0.94–1.30)	1.08(0.92–1.27)
≥140/90	505/1748	28.9	1.95(1.68–2.26)	1.82(1.57–2.12)	1.66(1.42–1.93)	1.56(1.32–1.83)
*p*_trend_		<.001	<.001	<.001	<.001	<.001
Prediabetes	920/4868	18.9				
<130/80	237/1667	14.2	1.00 (Reference)	1.00 (Reference)	1.00 (Reference)	1.00 (Reference)
130–139/80–89	262/1451	18.1	1.33(1.10–1.61)	1.30(1.07–1.58)	1.23(1.01–1.49)	1.19(0.98–1.45)
≥140/90	421/1750	24.1	1.91(1.60–2.28)	1.84(1.54–2.20)	1.66(1.39–1.99)	1.59(1.32–1.92)
*p*_trend_		<.001	<.001	<.001	<.001	<.001
Diabetes	535/2187	24.5				
<130/80	104/632	16.5	1.00 (Reference)	1.00 (Reference)	1.00 (Reference)	1.00 (Reference)
130–139/80–89	156/642	24.3	1.63(1.24–2.15)	1.62(1.23–2.15)	1.56(1.18–2.07)	1.51(1.14–2.01)
≥140/90	275/913	30.1	2.19(1.70–2.82)	2.14(1.66–2.77)	1.98(1.53–2.57)	1.89(1.44–2.46)
*p*_trend_		<.001	<.001	<.001	<.001	<.001

Abbreviations: BMI, body mass index; BP, blood pressure; CI, confidence interval; CKD, chronic kidney disease; RR, relative risk; TC, total cholesterol; TG, triglyceride.

^a^ *p*_interaction_ between glycemic categories and BP on CKD risk > .05.

^b^ Adjusted for age and sex.

^c^ Adjusted for age, sex, BMI, TC, and TG.

^d^ Adjusted for age, sex, BMI, TC, TG, antidiabetic medications, antihypertensive medications, physical activity, degree of education, smoking, and drinking status.

**FIGURE 1:**
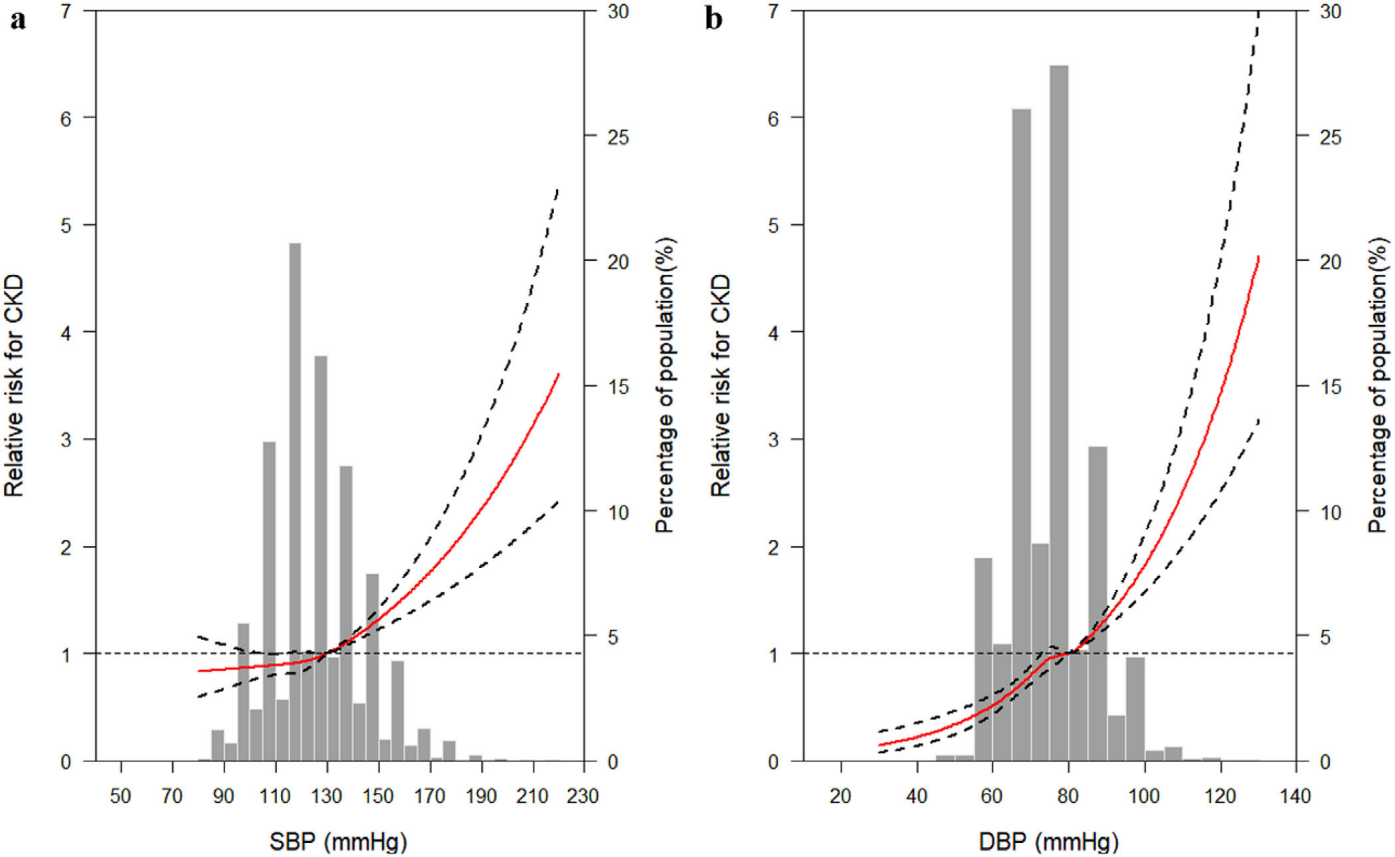
The RCS for the association between SBP (a), DBP (b), and incident CKD. The lines represent adjusted RR based on RCS for the continuous values of SBP and DBP, and lead in the logistic regression model. Knots were placed at the 20th, 40th, 60th, and 80th percentiles of the BP distribution, and the reference value was set at 130 mmHg for SBP and 80 mmHg for DBP. Adjustment factors were age, sex, BMI, TC, TG, antidiabetic medications, antihypertensive medications, physical activity, degree of education, smoking, and drinking status. Abbreviations: BMI, body mass index; BP, blood pressure; CKD, chronic kidney disease; DBP, diastolic blood pressure; RCS, restricted cubic spline regression; RR, relative risk; SBP, systolic blood pressure; TC, total cholesterol; TG, triglyceride

In Figure 1, RCS analysis indicated significant nonlinear associations for SBP and incident CKD (*p*_nonlinearity_ = .040), but the result of DBP was opposite (*p*_nonlinearity_ = .146). The risk of incident CKD was relatively flat until BP ≥ 130/80 mmHg and then started to increase rapidly afterwards.

**FIGURE 2:**
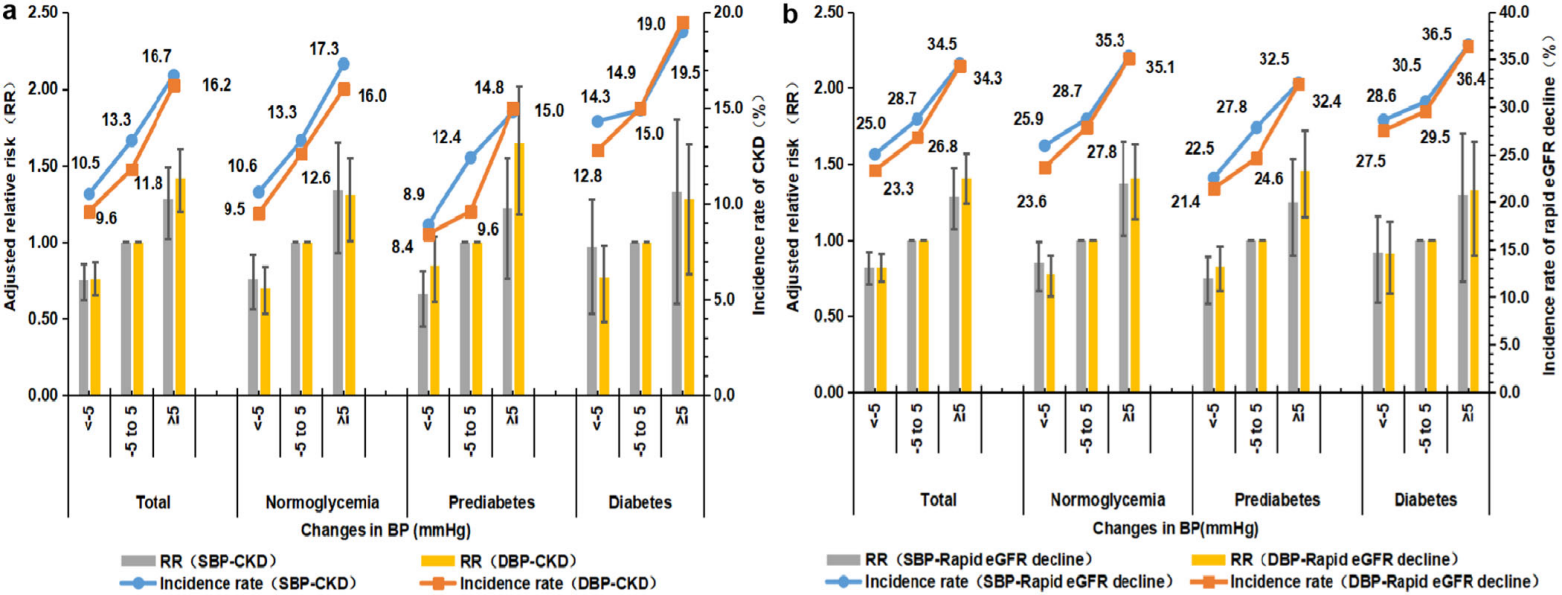
Adjusted RR (95% CI) and incidence rate for CKD (a) and rapid eGFR decline (b) according to changes in BP among participants of various blood glucose categories. Adjusted for age, sex, BMI, TC, TG, antidiabetic medications, antihypertensive medications, physical activity, degree of education, smoking, and drinking status. Abbreviations: BMI, body mass index; BP, blood pressure; CI, confidence interval; CKD, chronic kidney disease; DBP, diastolic blood pressure; eGFR, estimated glomerular filtration rate; RR, relative risk; SBP, systolic blood pressure; TC, total cholesterol; TG, triglyceride

Compared with individuals who were with stable SBP (−5 to 5 mmHg), the risk of incident CKD reduced when SBP decreased by more than 5 mmHg, and increased when SBP increased ≥5 mmHg among the whole participants, normoglycemia, and prediabetes participants, no significant association was observed in DM cases (Figure 2, Tables S6–S7). Similar findings were obtained for DBP. After exclusion of participants with antihypertensive or antidiabetic treatment, similar results were obtained except that the association between SBP levels change ≥5 mmHg and incident CKD risk (Tables S8–S9). There was a similar result when we constructed five categories of SBP or DBP changes as <−15, −15 to −5, −5 to 5, 5–15, and ≥15 mmHg (Tables S10–S11).

In sensitivity analyses, we further defined outcome as rapid eGFR decline among follow‐up of 2008–2013 (Table 3). During the about 5 years follow‐up period, there were 3506 (22.2%) developed rapid eGFR decline. Compared with those with BP < 130/80 mmHg, individuals with BP of 130–139/80–89 mmHg had increased risk of rapid eGFR decline among different glycemic categories. Intensive increased risk of rapid eGFR decline were observed in those with BP of 130–139/80–89 mmHg among the whole, normoglycemia, and prediabetes participants. Similar results were obtained between SBP or DBP changes and rapid eGFR decline risk (Figure 2, Tables S12–S13).

**TABLE 3:** Multivariable‐adjusted RR and 95% CI for the rapid eGFR decline risk according to different glycemic categories and BP categories (2008–2013)^a^

BP category (mmHg)	No./total	Rate	Crude RR (95% CI)	Adjusted RR (95% CI)^b^
Total	3506/15 781	22.2		
<130/80	927/5404	17.2	1.00 (Reference)	1.00 (Reference)
130–139/80–89	1022/4743	21.5	1.33(1.20–1.46)	1.34(1.21–1.48)
≥140/90	1557/5643	27.6	1.84(1.68–2.02)	1.86(1.69–2.04)
*p*_trend_		<.001	<.001	<.001
Normoglycemia	1789/7027	25.5		
<130/80	550/2695	20.4	1.00 (Reference)	1.00 (Reference)
130–139/80–89	550/2134	25.8	1.35(1.18–1.55)	1.39(1.21–1.59)
≥140/90	689/2198	31.3	1.78(1.56–2.03)	1.84(1.61–2.11)
*p*_trend_		<.001	<.001	<.001
Prediabetes	1112/5914	18.8		
<130/80	256/1929	13.3	1.00 (Reference)	1.00 (Reference)
130–139/80–89	322/1784	18.0	1.44(1.20–1.72)	1.39(1.16–1.66)
≥140/90	534/2201	24.3	2.09(1.78–2.47)	1.98(1.67–2.34)
*p*_trend_		<.001	<.001	<.001
Diabetes	605/2840	21.3		
<130/80	121/780	15.5	1.00 (Reference)	1.00 (Reference)
130–139/80–89	150/825	18.2	1.21(0.93–1.57)	1.18(0.91–1.54)
≥140/90	334/1235	27.0	2.02(1.60–2.54)	1.89(1.50–2.40)
*p*_trend_		<.001	<.001	<.001

Abbreviations: BMI, body mass index; BP, blood pressure; CI, confidence interval; eGFR, estimated glomerular filtration rate; RR, relative risk; TC, total cholesterol; TG, triglyceride.

^a^ *p*_interaction_ between glycemic categories and BP on the rapid eGFR decline > .05.

^b^ Adjusted for age, sex, BMI, TC, TG, antidiabetic medications, antihypertensive medications, physical activity, degree of education, smoking, and drinking status.

Subgroup analysis for incident CKD risk was performed according to baseline characteristics including age, sex, BMI, smoking status, drinking status, and baseline eGFR. No significant interaction was found between blood glucose and BP for CKD across groups for various baseline characteristics (all *p*_Interaction_ *> *.05) (Tables S14–S16).

## 4 DISCUSSION

In the present prospective study, we found that a BP of 130–139/80–89 mmHg defined by 2017 ACC/AHA hypertension guideline was associated with a significantly high risk of incident CKD and rapid eGFR decline in Chinese adults. Similar results were observed in individuals with prediabetes or DM. Meanwhile, when SBP and DBP decreases or increases by more than 5 mmHg, the risk of incident CKD decreases and increases accordingly. These associations were also observed in normoglycemia and prediabetes participants, but not in DM participants.

It is controversial whether BP levels of 130–139/80–89 mmHg were associated with CKD risk, especially after the 2017ACC/AHA guideline were published. Numerous studies failed to find a greater risk of CKD in association with stage 1 hypertension^10^, ^12^, ^27^; however, a meta‐analysis including 16 cohort studies (315 321 participants) showed that BP of 120–139/80–89 mmHg was continuously associated with higher risk of decreased eGFR levels in the general participants.^11^ Similarly, an analysis constituting nearly 267, 469 adults from Hong Kong suggested consistent association between repeated SBP of 125–134 mmHg and CKD risk.^13^ Although the different results may be attributed to the different cohort samples, follow‐up durations, and definitions of CKD, these findings suggest that the risk of CKD was higher even if BP is less than 140/90 mmHg. Recently, the Iki epidemiological Study of atheroSclerosis And Chronic Kidney Disease (ISSA‐CKD) with 3, 269 participants assessed the CKD risk based on the new BP definition, and the results showed that stage 1 hypertension had an increased CKD risk. However, these associations changed to null after using time‐averaged BP during follow‐up as exposure variable instead of baseline BP values.^28^ In 2017 guidelines, the ACC recommended that all adults with hypertension and CKD should be treated to a target BP of <130/80 mmHg regardless of proteinuria.^4^ But a meta‐analysis including nine trials with 8127 patients and a median follow‐up of 3.3 years showed that intensive BP control (<130/80 mmHg) did not provide additional benefit for renal outcomes in patients with CKD but without DM.^29^ Another study, used averaged BP, found that the relationship between BP ≥ 130/80 mmHg and increased CKD risk still existed in participant with DM.^30^ It can be seen that the association of stage 1 hypertension and CKD risk at different glycemic status was still unclear and short of comprehensive and systematic research.

Therefore, we combined with BP categories and glycemic status and found that stage 1 hypertension was significantly associated with a higher CKD risk in total, prediabetes, or DM participants. Our study lends the supports to the results of clinical trials among normoglycemia to a certain extent, and it may be more beneficial for CKD prevention and treatment when BP reaches stage 1 hypertension, particularly in prediabetes individuals with BMI < 24 kg/m^2^. At the same time, research shows that the presence of prediabetes did not appear to augment the risk for kidney events during intensive SBP lowering.^18^ Our results in prediabetes may provide a new target for early prevention and control of CKD. This result was not obvious when we divided BP levels into five groups, but RCS analysis indicated the risk of incident CKD was relatively flat until BP ≥ 130/80 mmHg and then started to increase rapidly afterwards. It is worth noting that individuals of BP ≥ 130/80 mmHg had increased risk of rapid eGFR decline no matter which category the blood glucose was in. These results may prompt that stage 1 hypertension can increase the risk of renal impairment and blood glucose status has a greater impact on BP and renal impairment. Of course, it may be closely related to the level of eGFR for participants at baseline, and the specific results need to be verified by further clinical trials.

As far as we know, a large majority of previous studies, which showed prehypertension of traditional definition was an independent risk factor for CKD development, examined only baseline BP as an exposure of interest and did not consider the dynamic changes of BP over time and blood glucose status. Lately, a prospective community‐based cohort study with 4643 individuals from Korean pointed out increasing SBP over time without reaching the hypertension threshold is associated with a significantly increased risk of incident CKD in healthy adults compared with stable trajectory.^20^ In the present study, the analysis on SBP or DBP change among normoglycemia and prediabetes found that an increase in SBP or DBP (>5 mmHg) was associated with higher risk of CKD and rapid eGFR decline, and a decrease in SBP or DBP (>5 mmHg) reduced more than 20% risk of CKD and rapid GFR decline. It shows that it is important to pay attention to the long‐term changes of BP for the prevention of CKD. Although some randomized controlled trials showed that BP reduction < 120 mmHg resulted in more CKD events, lowering BP significantly decreased albuminuria.^31^, ^32^ The corresponding relationship was not obvious in DM participants in the present study, relatively limited sample size of diabetic patients might contribute to these findings. But recently it was reported that the effect of simple hypertension or simple diabetes on new‐onset CKD was not significantly different.^33^ Meanwhile, no significant interaction was found between blood glucose and BP on CKD risk, so DM may have great impact on CKD, and hyperglycemia status with long duration may have an irreversible effect on renal function when both diabetes and hypertension existed simultaneously.

Our study had several strengths. First of all, the prospective cohort study design and the long follow‐up period were useful to determine the association of different BP and blood glucose categories with the incident CKD risk. Moreover, we evaluated the associations of BP levels with short‐term and long‐term risk of incident CKD and further assessed the associations of BP change over time with CKD risk, which was more systematic and comprehensive than previous studies. In addition, we performed a series of subgroup and sensitivity analyses, taking into account as many confounding factors as possible, all of which obtained similar results, indicating that our findings were robust.

However, some limitations should be considered. Firstly, since we did not collect detailed data on the types and dosage of antihypertensive and antidiabetic medications, we were unable to analyze the impact of different antihypertensive and antidiabetic medications on the results. Meanwhile, the duration of hypertension and DM were not taken into consideration, and we classified different glycemia categories only using single point FBG level, but not glycosylated hemoglobin (HbA1c), which may affect the findings. But some cohort study found that the duration of DM and the HbA1c levels were not related to the renal outcome in the 4‐year follow‐up for patients with DM.^34^ Secondly, the new‐onset CKD was based on the new occurrence of eGFR < 60 ml/min/1.73 m^2^ in the follow‐ups so participants with acute kidney injury were also included in our study. However, it might have little effect on the results because there was only a small part of participants with acute kidney injury among middle‐aged and elderly people,^33^ in particular, kidney disease was excluded at baseline. In addition, in the present study, we did not collect urinary albumin data, which would lead to false negative for CKD with an eGFR above 60 ml/min/1.73 m^2^. Thirdly, we measured BP levels only once at baseline and follow‐ups, which might misclassify the BP group. More studies were warranted to validate our findings. Fourthly, our analyses were restricted to the middle‐aged and elderly Chinese participants and thus may not be generalized to other age groups or ethnicities. Fifthly, we could not explore the impact of time effect on the results because the time record of CKD incidence could not be very accurate by follow‐up time of every 5 years. Finally, although a range of potential confounding factors were adjusted in multivariate analysis, we still could not eliminate the unmeasured and residual confounding.

## 5 CONCLUSIONS

The present study found that the BP of 130–139/80–89 mmHg combined with prediabetes or DM had an increased risk of incident CKD and rapid eGFR decline in Chinese adults. The long‐term changes of BP by more than 5 mmHg among normoglycemia or prediabetes were associated with the risk of incident CKD and rapid eGFR decline.

## AUTHOR CONTRIBUTIONS

Jia He and Meian He had full access to all of the data in the study and take responsibility for the integrity of the data and the accuracy of the data analysis. Jia He and Meian He contributed to conception, design, and write the manuscript. Jia He, Zhaoyang Li, Ruixin Wang, Hongli Nie, and Fei Wang contributed to analysis and interpretation of the data. Jing Yuan, Xiaoping Miao, Ping Yao, Sheng Wei, Xiaomin Zhang, Huan Guo, Handong Yang, Tangchun Wu, and Meian He contributed to collection and assembly of data. All authors reviewed/edited the manuscript for important intellectual content. All authors read and approved the final manuscript.

## Supporting information

Supporting information.
