# Supplementary material for: Association of blood pressure and long‐term change with chronic kidney disease risk among Chinese adults with different glucose metabolism according to the 2017 ACC/AHA guidelines
Source: J Clin Hypertens (Greenwich). 2021 Nov 12;23(12):2080–8. doi: 10.1111/jch.14371 (PMC8696214; doi:10.1111/jch.14371)
Supplement: Supplementary file 1 — Supporting information. [file JCH-23-2080-s001.doc]

**Supporting Information for online publication only**

**Association of blood pressure and long-term change with chronic kidney disease risk among Chinese adults with different glucose metabolism according to the 2017 ACC/AHA guidelines**

Jia He MD, PhD^1^; Zhaoyang Li MD, PhD^1^; Ruixin Wang MD, PhD^1^; Hongli Nie MM^1^; Fei Wang MD, PhD^1^; Jing Yuan MD, PhD^1^; Xiaoping Miao MD, PhD^1^; Ping Yao MD, PhD^1^; Sheng Wei MD, PhD^1^; Xiaomin Zhang MD, PhD^1^; Huan Guo MD, PhD^1^; Handong Yang MD, PhD^2^; Tangchun Wu MD, PhD^1^; Meian He MD, PhD^1^**^†^**

**Author affiliations**

^1^Department of Occupational and Environmental Health and State Key Laboratory of Environmental Health for Incubating, School of Public Health, Tongji Medical College, Huazhong University of Science and Technology, Wuhan, Hubei, China; ^2^Dongfeng Central Hospital, Dongfeng Motor Corporation and Hubei University of Medicine, Shiyan, Hubei, China.

**^†^Corresponding author:** Meian He

Department of Occupational and Environmental Health and State Key Laboratory of Environmental Health for Incubating, School of Public Health, Tongji Medical College, Huazhong University of Science and Technology, 13 Hangkong Rd, Wuhan, Hubei, 430030, China. Tel: 86-27-83692882; Fax: 86-27-83692560; E-mail: [hemeian@hotmail.com](mailto:hemeian@hotmail.com)

**Running title:** CKD Risk on BP Combined with Glucose Metabolism

**Contents**

**Figure S1.** Flow chart of the participants in baseline blood pressure analysis.

**Figure S2.** Flow chart of the participants in longitudinal blood pressure analysis.

**Table S1.** Multivariable-adjusted RR of incident CKD according to different blood glucose categories and BP categories (2008-2013).

**Table S2.** Multivariable-adjusted RR for the risks of incident CKD according to different glycemic categories and BP categories among participants without antihypertensive or antidiabetic medications at baseline (2008-2018).

**Table S3.** Multivariable-adjusted RR for the risks of incident CKD according to different glycemic categories and BP categories among participants without antihypertensive or antidiabetic medications at baseline (2008-2013).

**Table S4.** Multivariable-adjusted RR for the risks of incident CKD according to different blood glucose categories and BP categories (2008-2018) (5 categories of BP).

**Table S5.** Multivariable-adjusted RR for the risks of incident CKD according to different blood glucose categories and BP categories (2008-2013) (5 categories of BP).

**Table S6.** Adjusted RR and 95% CI for CKD risk according to changes in SBP among participants of various blood glucose categories.

**Table S7.** Adjusted RR and 95% CI for CKD risk according to changes in DBP among participants of various blood glucose categories.

**Table S8.** Adjusted RR and 95% CI for CKD risk according to changes in SBP among participants of various blood glucose categories without antihypertensive or antidiabetic medications at baseline.

**Table S9.** Adjusted RR and 95% CI for CKD risk according to changes in DBP among participants of various blood glucose categories without antihypertensive or antidiabetic medications at baseline.

**Table S10.** Adjusted RR and 95% CI for CKD risk according to changes in SBP among participants of various blood glucose categories (5 categories of changes in SBP).

**Table S11.** Adjusted RR and 95% CI for CKD risk according to changes in DBP among participants of various blood glucose categories (5 categories of changes in DBP).

**Table S12.** Adjusted RR and 95% CI for rapid eGFR decline risk according to changes in SBP among participants of various blood glucose categories.

**Table S13.** Adjusted RR and 95% CI for rapid eGFR decline risk according to changes in DBP among participants of various blood glucose categories.

**Table S14.** Subgroup analysis of associations between BP categories and risk of incident CKD among participants of various blood glucose categories.

**Table S15.** Subgroup analysis of associations between changes in SBP and risk of incident CKD among participants of various blood glucose categories.

**Table S16.** Subgroup analysis of associations between changes in DBP and risk of incident CKD among participants of various blood glucose categories.


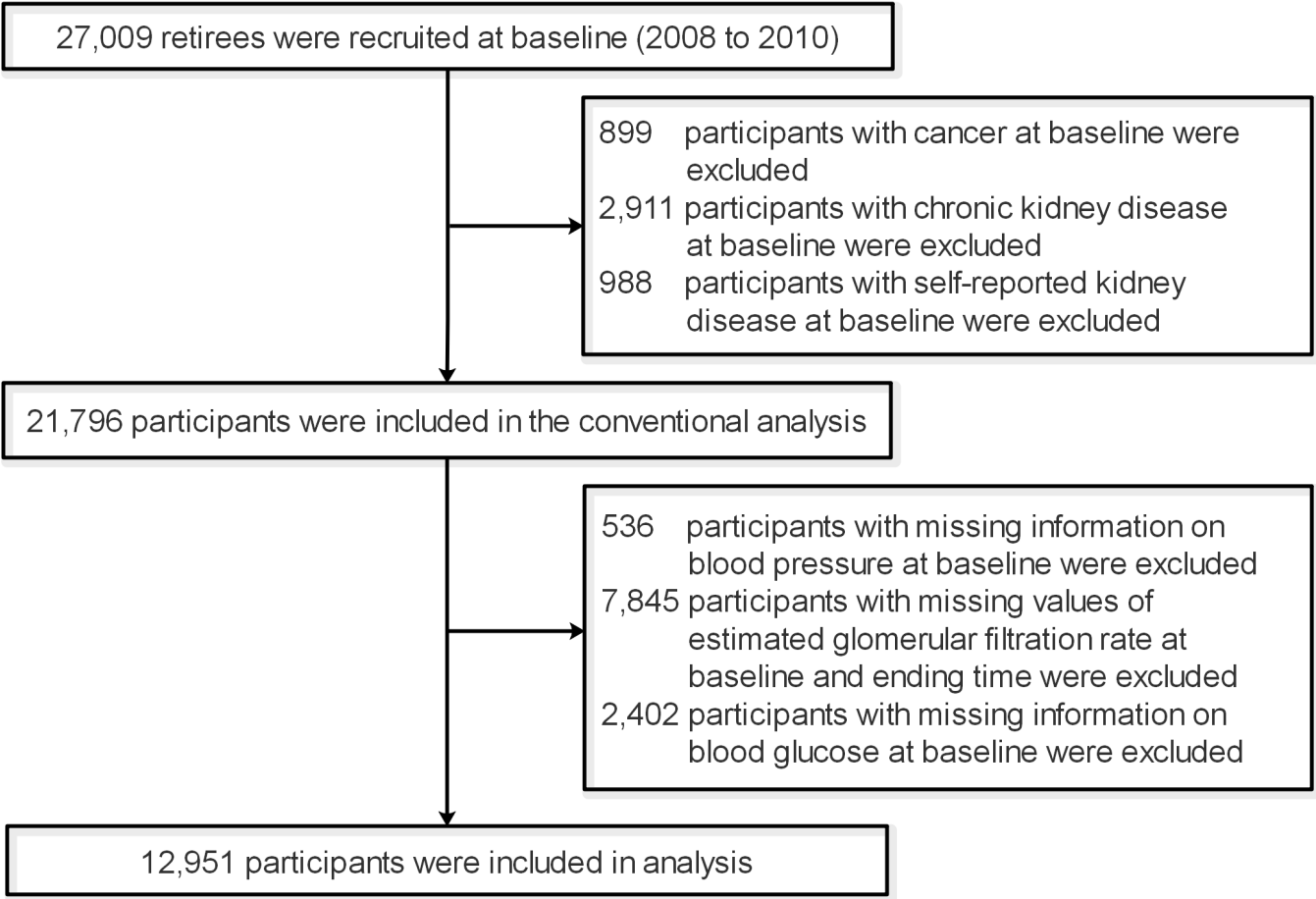


**Figure S1. Flow chart of the participants in baseline blood pressure analysis.**


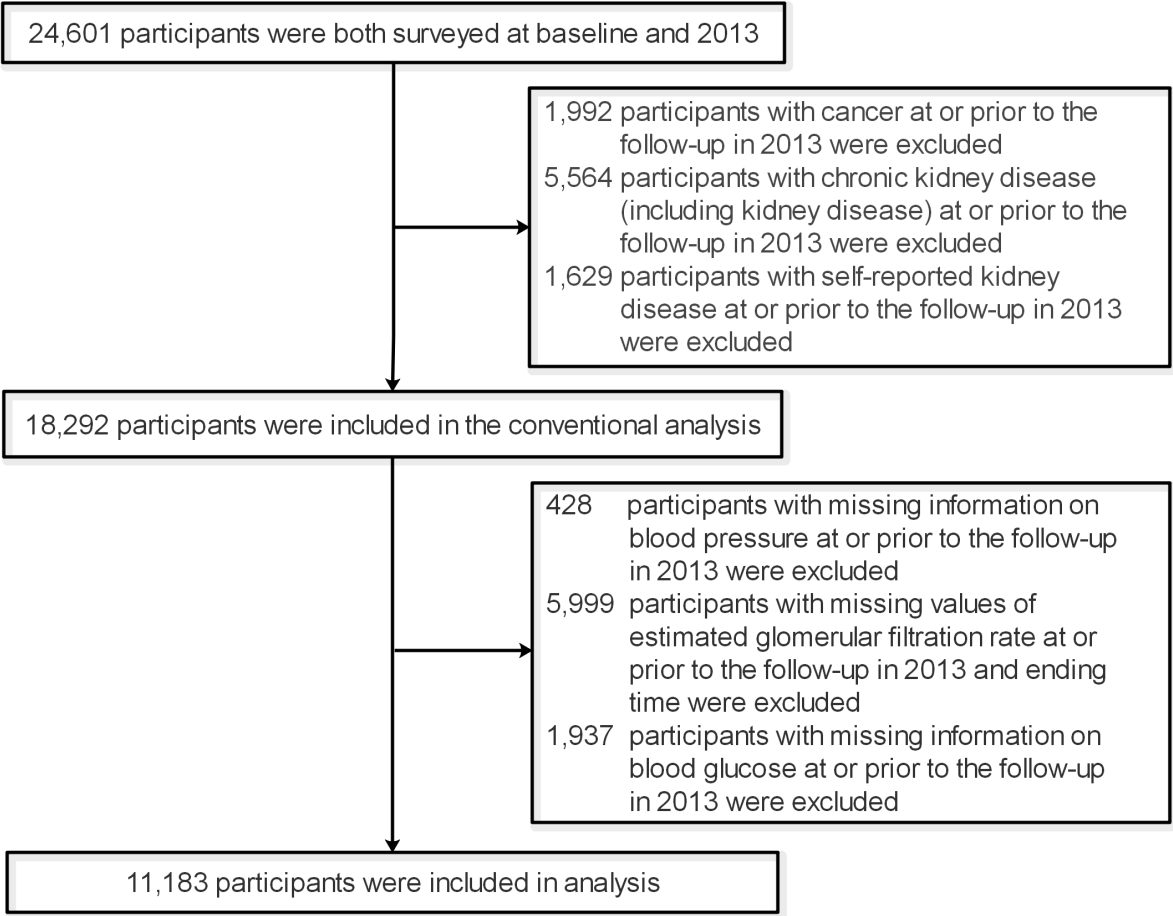


**Figure S2. Flow chart of the participants in longitudinal blood pressure analysis.**

| **Table S1.Multivariable-adjusted RR of incident CKD according to different blood glucose categories and BP categories (2008-2013)** | | | | | | |
| --- | --- | --- | --- | --- | --- | --- |
|  |  |  |  |  |  |  |
| BP category (mmHg) | n/total | Rate | Crude RR (95% CI) | Adjusted RR (95% CI) | | |
|  |  |  |  | Model 1^a^ | Model 2^b^ | Model 3^c^ |
| Total | 1947/15781 | 12.3 |  |  |  |  |
| <130/80 | 488/5404 | 9.0 | 1.00 (Reference) | 1.00 (Reference) | 1.00 (Reference) | 1.00 (Reference) |
| 130–139/80-89 | 534/4743 | 11.3 | 1.28(1.12-1.46) | 1.24(1.08-1.41) | 1.18(1.03-1.34) | 1.16(1.02-1.33) |
| ≥140/90 | 925/5634 | 16.4 | 1.98(1.76-2.22) | 1.84(1.64-2.07) | 1.68(1.49-1.89) | 1.62(1.42-1.83) |
| *P*_trend_ |  | <0.001 | <0.001 | <0.001 | <0.001 | <0.001 |
| Normoglycemia | 876/7027 | 12.5 |  |  |  |  |
| <130/80 | 272/2695 | 10.1 | 1.00 (Reference) | 1.00 (Reference) | 1.00 (Reference) | 1.00 (Reference) |
| 130–139/80-89 | 234/2134 | 11.0 | 1.10(0.81-1.32) | 1.06(0.88-1.28) | 1.03(0.85-1.24) | 1.03(0.85-1.25) |
| ≥140/90 | 370/2198 | 16.8 | 1.80(1.52-2.13) | 1.66(1.40-1.97) | 1.54(1.29-1.85) | 1.50(1.25-1.81) |
| *P*_trend_ |  | <0.001 | <0.001 | <0.001 | <0.001 | <0.001 |
| Prediabetes | 662/5914 | 11.2 |  |  |  |  |
| <130/80 | 139/1929 | 7.2 | 1.00 (Reference) | 1.00 (Reference) | 1.00 (Reference) | 1.00 (Reference) |
| 130–139/80-89 | 196/1784 | 11.0 | 1.59(1.27-2.00) | 1.55(1.23-1.95) | 1.46(1.16-1.84) | 1.45(1.14-1.83) |
| ≥140/90 | 327/2201 | 14.9 | 2.25(1.82-2.77) | 2.14(1.73-2.64) | 1.93(1.55-2.39) | 1.87(1.50-2.33) |
| *P*_trend_ |  | <0.001 | <0.001 | <0.001 | <0.001 | <0.001 |
| Diabetes | 409/2840 | 14.4 |  |  |  |  |
| <130/80 | 77/780 | 9.9 | 1.00 (Reference) | 1.00 (Reference) | 1.00 (Reference) | 1.00 (Reference) |
| 130–139/80-89 | 104/825 | 12.6 | 1.32(0.96-1.80) | 1.28(0.93-1.75) | 1.24(0.90-1.71) | 1.19(0.86-1.64) |
| ≥140/90 | 228/1235 | 18.5 | 2.07(1.57-2.72) | 1.98(1.50-2.62) | 1.81(1.37-2.41) | 1.70(1.27-2.27) |
| *P*_trend_ |  | <0.001 | <0.001 | <0.001 | <0.001 | <0.001 |
| Abbreviation: BP, blood pressure; RR, relative risk; CI, confidence interval; CKD, chronic kidney disease; BMI, body mass index; TC, total cholesterol; TG, triglyceride.  ^a^ adjusted for age and sex.  ^b^ adjusted for age, sex, BMI, TC, and TG.  ^c^ adjusted for age, sex, BMI, TC, TG, antidiabetic medications, antihypertensive medications, physical activity, degree of education, smoking and drinking status. | | | | | | |
|  |  |  |  |  |  |  |

| **Table S2.Multivariable-adjusted RR for the risks of incident CKD according to different glycemic categories and BP categories among participants without antihypertensive or antidiabetic medications at baseline (2008-2018)** | | | | |
| --- | --- | --- | --- | --- |
|
| BP category (mmHg) | n/total | Rate | Crude RR (95% CI) | Adjusted RR (95% CI)^a^ |
|
| Total | 1616/8981 | 15.0 |  |  |
| < 130/80 | 579/3868 | 17.9 | 1.00 (Reference) | 1.00 (Reference) |
| 130–139/80-89 | 483/2691 | 22.9 | 1.24(1.09-1.42) | 1.20(1.05-1.38) |
| ≥140/90 | 554/2422 | 18.0 | 1.69(1.48-1.92) | 1.57(1.37-1.80) |
| *P*_trend_ |  | <0.001 | <0.001 | <0.001 |
| Normoglycemia | 896/4695 | 19.1 |  |  |
| < 130/80 | 357/2171 | 16.4 | 1.00 (Reference) | 1.00 (Reference) |
| 130–139/80-89 | 262/1426 | 18.4 | 1.14(0.96-1.36) | 1.13(0.94-1.36) |
| ≥140/90 | 277/1098 | 25.2 | 1.71(1.44-2.05) | 1.63(1.35-1.96) |
| *P*_trend_ |  | <0.001 | <0.001 | <0.001 |
| Prediabetes | 594/3525 | 16.9 |  |  |
| < 130/80 | 195/1435 | 13.6 | 1.00 (Reference) | 1.00 (Reference) |
| 130–139/80-89 | 184/1047 | 17.6 | 1.36(1.09-1.69) | 1.31(1.05-1.65) |
| ≥140/90 | 215/1043 | 20.6 | 1.65(1.34-2.04) | 1.52(1.22-1.90) |
| *P*_trend_ |  | <0.001 | <0.001 | 0.001 |
| Diabetes | 126/761 | 16.6 |  |  |
| < 130/80 | 27/262 | 10.3 | 1.00 (Reference) | 1.00 (Reference) |
| 130–139/80-89 | 37/218 | 17.0 | 1.78(1.05-3.03) | 1.88(1.09-3.26) |
| ≥140/90 | 62/281 | 22.1 | 2.46(1.51-4.01) | 2.36(1.42-3.92) |
| *P*_trend_ |  | <0.001 | 0.001 | 0.004 |
| Abbreviation: BP, blood pressure; RR, relative risk; CI, confidence interval; CKD, chronic kidney disease; BMI, body mass index; TC, total cholesterol; TG, triglyceride.  ^a^ Adjusted for age, sex, BMI, TC, TG, physical activity, degree of education, smoking and drinking status. | | | | |
|

| **Table S3. Multivariable-adjusted RR for the risks of incident CKD according to different glycemic categories and BP categories among participants without antihypertensive or antidiabetic medications at baseline (2008-2013)** | | | | |
| --- | --- | --- | --- | --- |
|
| BP category (mmHg) | n/total | Rate | Crude RR (95% CI) | Adjusted RR (95% CI)^a^ |
|
| Total | 1121/10775 | 10.4 |  |  |
| < 130/80 | 367/4409 | 8.3 | 1.00 (Reference) | 1.00 (Reference) |
| 130–139/80-89 | 323/3283 | 9.8 | 1.20(1.03-1.41) | 1.16(0.99-1.37) |
| ≥140/90 | 431/3084 | 14.0 | 1.79(1.54-2.01) | 1.64(1.41-1.92) |
| *P*_trend_ |  | <0.001 | <0.001 | <0.001 |
| Normoglycemia | 608/5552 | 11.0 |  |  |
| < 130/80 | 232/2450 | 9.5 | 1.00 (Reference) | 1.00 (Reference) |
| 130–139/80-89 | 169/1706 | 9.9 | 1.05(0.85-1.30) | 1.05(0.84-1.30) |
| ≥140/90 | 207/1396 | 14.8 | 1.66(1.36-2.03) | 1.57(1.27-1.95) |
| *P*_trend_ |  | <0.001 | <0.001 | <0.001 |
| Prediabetes | 407/4247 | 9.6 |  |  |
| < 130/80 | 109/1647 | 6.6 | 1.00 (Reference) | 1.00 (Reference) |
| 130–139/80-89 | 125/1289 | 9.7 | 1.52(1.16-1.98) | 1.44(1.09-1.90) |
| ≥140/90 | 173/1311 | 13.2 | 2.15(1.67-2.76) | 1.95(1.50-2.53) |
| *P*_trend_ |  | <0.001 | <0.001 | <0.001 |
| Diabetes | 106/976 | 10.9 |  |  |
| < 130/80 | 26/312 | 8.3 | 1.00 (Reference) | 1.00 (Reference) |
| 130–139/80-89 | 29/287 | 10.1 | 1.24(0.71-2.16) | 1.22(0.69-2.17) |
| ≥140/90 | 51/377 | 13.5 | 1.72(1.05-2.83) | 1.63(0.96-2.74) |
| *P*_trend_ |  | 0.028 | <0.001 | 0.171 |
| Abbreviation: BP, blood pressure; RR, relative risk; CI, confidence interval; CKD, chronic kidney disease; BMI, body mass index; TC, total cholesterol; TG, triglyceride.  ^a^ Adjusted for age, sex, BMI, TC, TG, physical activity, degree of education, smoking and drinking status. | | | | |
|

| **Table S4. Multivariable-adjusted RR for the risks of incident CKD according to different blood glucose categories and BP categories (2008-2018) (5 categories of BP)** | | | | | | |
| --- | --- | --- | --- | --- | --- | --- |
|  |  |  |  |  |  |  |
| BP category (mmHg) | n/total | Rate | Crude RR (95% CI) | Adjusted RR (95% CI) | | |
|  |  |  |  | Model 1^a^ | Model 2^b^ | Model 3^c^ |
| Total | 2727/12951 | 21.1 |  |  |  |  |
| <120/80 | 420/2780 | 15.1 | 1.00 (Reference) | 1.00 (Reference) | 1.00 (Reference) | 1.00 (Reference) |
| 120–129/<80 | 330/1887 | 17.5 | 1.19(1.02-1.40) | 1.06(0.90-1.24) | 1.03(0.87-1.21) | 0.99(0.84-1.17) |
| 130–139/80-89 | 776/3873 | 20.0 | 1.41(1.24-1.60) | 1.31(1.15-1.49) | 1.22(1.06-1.39) | 1.18(1.03-1.35) |
| 140–159/90-99 | 778/3142 | 24.8 | 1.85(1.62-2.11) | 1.66(1.45-1.90) | 1.50(1.31-1.72) | 1.41(1.23-1.62) |
| ≥160/100 | 423/1269 | 33.3 | 2.81(2.40-3.28) | 2.55(2.17-2.99) | 2.24(1.91-2.64) | 2.11(1.78-2.49) |
| *P*_trend_ |  | <0.001 | <0.001 | <0.001 | <0.001 | <0.001 |
| Normoglycemia | 1272/5896 | 21.6 |  |  |  |  |
| <120/80 | 248/1542 | 16.1 | 1.00 (Reference) | 1.00 (Reference) | 1.00 (Reference) | 1.00 (Reference) |
| 120–129/<80 | 161/826 | 19.5 | 1.26(1.01-1.57) | 1.12(0.89-1.40) | 1.10(0.88-1.38) | 1.06(0.84-1.33) |
| 130–139/80-89 | 358/1780 | 20.1 | 1.31(1.10-1.57) | 1.23(1.03-1.47) | 1.15(0.96-1.38) | 1.11(0.92-1.34) |
| 140–159/90-99 | 327/1242 | 26.3 | 1.87(1.55-2.25) | 1.68(1.39-2.03) | 1.52(1.25-1.85) | 1.42(1.16-1.73) |
| ≥160/100 | 178/506 | 35.2 | 2.83(2.26-3.56) | 2.52(2.00-3.18) | 2.26(1.79-2.87) | 2.09(1.63-2.67) |
| *P*_trend_ |  | <0.001 | <0.001 | <0.001 | <0.001 | <0.001 |
| Prediabetes | 920/4868 | 18.9 |  |  |  |  |
| <120/80 | 122/917 | 13.3 | 1.00 (Reference) | 1.00 (Reference) | 1.00 (Reference) | 1.00 (Reference) |
| 120–129/<80 | 115/750 | 15.3 | 1.18(0.90-1.55) | 1.08(0.81-1.42) | 1.06(0.80-1.40) | 1.06(0.80-1.40) |
| 130–139/80-89 | 262/1451 | 18.1 | 1.43(1.14-1.81) | 1.34 (1.06-1.70) | 1.27(1.00-1.61) | 1.27(1.00-1.62) |
| 140–159/90-99 | 272/1256 | 21.7 | 1.80(1.43-2.27) | 1.65(1.30-2.09) | 1.50(1.18-1.91) | 1.49(1.16-1.90) |
| ≥160/100 | 149/494 | 30.2 | 2.81(2.15-3.69) | 2.66(2.02-3.50) | 2.34(1.77-3.09) | 2.30(1.72-3.06) |
| *P*_trend_ |  | <0.001 | <0.001 | <0.001 | <0.001 | <0.001 |
| Diabetes | 535/2187 | 24.5 |  |  |  |  |
| <120/80 | 50/321 | 15.6 | 1.00 (Reference) | 1.00 (Reference) | 1.00 (Reference) | 1.00 (Reference) |
| 120–129/<80 | 54/311 | 17.4 | 1.14(0.75-1.74) | 1.00(0.65-1.53) | 0.93(0.60-1.43) | 0.88(0.57-1.35) |
| 130–139/80-89 | 156/642 | 24.3 | 1.74(1.22-2.47) | 1.62(1.13-2.31) | 1.50(1.05-2.16) | 1.39(0.97-2.00) |
| 140–159/90-99 | 179/644 | 27.8 | 2.09(1.47-2.95) | 1.90(1.34-2.70) | 1.71(1.20-2.45) | 1.54(1.07-2.22) |
| ≥160/100 | 96/269 | 35.7 | 3.01(2.03-4.45) | 2.78(1.87-4.13) | 2.42(1.62-3.62) | 2.17(1.44-3.28) |
| *P*_trend_ |  | <0.001 | <0.001 | <0.001 | <0.001 | <0.001 |
| Abbreviation: BP, blood pressure; RR, relative risk; CI, confidence interval; CKD, chronic kidney disease; BMI, body mass index; TC, total cholesterol; TG, triglyceride.  ^a^ adjusted for age and sex.  ^b^ adjusted for age, sex, BMI, TC, and TG.  ^c^ adjusted for age, sex, BMI, TC, TG, antidiabetic medications, antihypertensive medications, physical activity, degree of education, smoking and drinking status. | | | | | | |
|  |  |  |  |  |  |  |

| **Table S5. Multivariable-adjusted RR** for the risks of **incident** CKD according to **different blood glucose categories and BP categories** (2008-2013) **(5 categories of BP)** | | | | | | |
| --- | --- | --- | --- | --- | --- | --- |
|
|  |
|
| BP category (mmHg) | n/total | Rate | Crude RR (95% CI) | Adjusted RR (95% CI) | | |
| Model 1^a^ | Model 2^b^ | Model 3^c^ |
| Total | 1947/15781 | 12.3 |  |  |  |  |
| <120/80 | 284/3192 | 8.9 | 1.00 (Reference) | 1.00 (Reference) | 1.00 (Reference) | 1.00 (Reference) |
| 120–129/<80 | 204/2212 | 9.2 | 1.04(0.86 -1.26) | 0.91(0.75-1.11) | 0.89(0.73-1.08) | 0.88(0.73-1.07) |
| 130–139/80-89 | 534/4743 | 11.3 | 1.30(1.12-1.51) | 1.19(1.02-1.39) | 1.12(0.96-1.30) | 1.10(0.94-1.29) |
| 140–159/90-99 | 586/3979 | 14.7 | 1.77(1.52-2.06) | 1.55(1.33-1.80) | 1.40(1.20-1.64) | 1.35(1.15-1.58) |
| ≥160/100 | 339/1655 | 20.5 | 2.64(2.22-3.13) | 2.36(1.98-2.80) | 2.09(1.75-2.50) | 1.98(1.65-2.38) |
| *P*_trend_ |  | <0.001 | <0.001 | <0.001 | <0.001 | <0.001 |
| Normoglycemia | 876/7027 | 12.5 |  |  |  |  |
| <120/80 | 172/1739 | 9.9 | 1.00 (Reference) | 1.00 (Reference) | 1.00 (Reference) | 1.00 (Reference) |
| 120–129/<80 | 100/956 | 10.5 | 1.06(0.82-1.38) | 0.95(0.73-1.23) | 0.95(0.72-1.23) | 0.94(0.72-1.23) |
| 130–139/80-89 | 234/2134 | 11.0 | 1.12(0.91-1.38) | 1.04(0.84-1.29) | 1.02(0.83-1.26) | 1.01(0.81-1.25) |
| 140–159/90-99 | 237/1532 | 15.5 | 1.67(1.35-2.06) | 1.47(1.19-1.82) | 1.42(1.14-1.76) | 1.35(1.07-1.68) |
| ≥160/100 | 133/666 | 20.0 | 2.27(1.78-2.91) | 2.01(1.56-2.59) | 1.90(1.47-2.46) | 1.79(1.37-2.35) |
| *P*_trend_ |  | <0.001 | <0.001 | <0.001 | <0.001 | <0.001 |
| Prediabetes | 662/5914 | 11.2 |  |  |  |  |
| <120/80 | 77/1060 | 7.3 | 1.00 (Reference) | 1.00 (Reference) | 1.00 (Reference) | 1.00 (Reference) |
| 120–129/<80 | 62/869 | 7.1 | 0.98(0.69-1.39) | 0.86(0.60-1.22) | 0.84(0.59-1.20) | 0.83(0.58-1.19) |
| 130–139/80-89 | 196/1784 | 11.0 | 1.58(1.20-2.08) | 1.44(1.09-1.91) | 1.34(1.02-1.78) | 1.33(1.00-1.77) |
| 140–159/90-99 | 208/1579 | 13.2 | 1.94(1.47-2.55) | 1.72(1.30-2.27) | 1.55(1.17-2.05) | 1.50(1.13-2.00) |
| ≥160/100 | 119/622 | 19.1 | 3.02(2.22-4.10) | 2.74(2.01-3.74) | 2.40(1.75-3.29) | 2.28(1.65-3.15) |
| *P*_trend_ |  | <0.001 | <0.001 | <0.001 | <0.001 | <0.001 |
| Diabetes | 409/2840 | 14.4 |  |  |  |  |
| <120/80 | 35/393 | 8.9 | 1.00 (Reference) | 1.00 (Reference) | 1.00 (Reference) | 1.00 (Reference) |
| 120–129/<80 | 42/387 | 10.9 | 1.25(0.78-1.00) | 1.12(0.69-1.81) | 1.06(0.65-1.72) | 1.06(0.65-1.72) |
| 130–139/80-89 | 104/825 | 12.6 | 1.48(0.99-2.21) | 1.36(0.90-2.04) | 1.28(0.85-1.94) | 1.27(0.84-1.93) |
| 140–159/90-99 | 141/868 | 16.2 | 1.98(1.34-2.93) | 1.78(1.20-2.65) | 1.60(1.07-2.39) | 1.58(1.06-2.37) |
| ≥160/100 | 87/367 | 23.7 | 3.18(2.08-4.85) | 2.95(1.92-4.52) | 2.63(1.70-4.07) | 2.63(1.70-4.08) |
| *P*_trend_ |  | <0.001 | <0.001 | <0.001 | <0.001 | <0.001 |
| Abbreviation: BP, blood pressure; RR, relative risk; CI, confidence interval; CKD, chronic kidney disease; BMI, body mass index; TC, total cholesterol; TG, triglyceride.  ^a^ adjusted for age and sex.  ^b^ adjusted for age, sex, BMI, TC, and TG.  ^c^ adjusted for age, sex, BMI, TC, TG, antidiabetic medications, antihypertensive medications, physical activity, degree of education, smoking and drinking status. | | | | | | |
|

| **Table S6. Adjusted RR and 95% CI for CKD risk according to changes in SBP among participants of various blood glucose categories** | | | | | | |
| --- | --- | --- | --- | --- | --- | --- |
|  |  |  |  |  |  |  |
| Changes in SBP(mmHg) | n/total | Rate | Crude RR (95% CI) | Adjusted RR (95% CI) | | |
|  |  |  |  | Model 1^a^ | Model 2^b^ | Model 3^c^ |
| Total | 1355/11183 | 12.1 |  |  |  |  |
| <-5 | 773/7333 | 10.5 | 0.77(0.66-0.90) | 0.74(0.63-0.87) | 0.74(0.63-0.87) | 0.75(0.64-0.88) |
| -5 to 5 | 233/1758 | 13.3 | 1.00 (Reference) | 1.00 (Reference) | 1.00 (Reference) | 1.00 (Reference) |
| ≥5 | 349/2092 | 16.7 | 1.31(1.10-1.57) | 1.30(1.08-1.55) | 1.28(1.07-1.54) | 1.28(1.07-1.54) |
| *P*_trend_ |  | <0.001 | <0.001 | <0.001 | <0.001 | <0.001 |
| Normoglycemia | 616/4970 | 12.4 |  |  |  |  |
| <-5 | 337/3167 | 10.6 | 0.77(0.62-0.97) | 0.74(0.59-0.93) | 0.75(0.59-0.94) | 0.76(0.60-0.96) |
| -5 to 5 | 112/840 | 13.3 | 1.00 (Reference) | 1.00 (Reference) | 1.00 (Reference) | 1.00 (Reference) |
| ≥5 | 167/963 | 17.3 | 1.36(1.05-1.77) | 1.36(1.04-1.76) | 1.33(1.03-1.74) | 1.34(1.03-1.75) |
| *P*_trend_ |  | 0.004 | <0.001 | <0.001 | <0.001 | <0.001 |
| Prediabetes | 456/4361 | 10.5 |  |  |  |  |
| <-5 | 262/2947 | 8.9 | 0.69(0.53-0.90) | 0.67(0.51-0.88) | 0.66(0.51-0.87) | 0.66(0.51-0.87) |
| -5 to 5 | 79/637 | 12.4 | 1.00 (Reference) | 1.00 (Reference) | 1.00 (Reference) | 1.00 (Reference) |
| ≥5 | 115/777 | 14.8 | 1.23(0.90-1.67) | 1.23(0.90-1.68) | 1.22(0.89-1.66) | 1.22(0.89-1.68) |
| *P*_trend_ |  | 0.063 | <0.001 | <0.001 | <0.001 | <0.001 |
| Diabetes | 283/1852 | 15.3 |  |  |  |  |
| <-5 | 174/1219 | 14.3 | 0.95(0.66-1.37) | 0.93(0.65-1.35) | 0.95(0.66-1.38) | 0.97(0.66-1.41) |
| -5 to 5 | 42/281 | 14.9 | 1.00 (Reference) | 1.00 (Reference) | 1.00 (Reference) | 1.00 (Reference) |
| ≥5 | 67/352 | 19.0 | 1.34(0.88-2.04) | 1.30(0.85-1.99) | 1.34(0.87-2.05) | 1.33(0.86-2.06) |
| *P*_trend_ |  | 0.117 | 0.092 | 0.117 | 0.107 | 0.142 |
| Abbreviation: SBP, systolic blood pressure; RR, relative risk; CI, confidence interval; CKD, chronic kidney disease; BMI, body mass index; TC, total cholesterol; TG, triglyceride.  ^a^ adjusted for age and sex.  ^b^ adjusted for age, sex, BMI, TC, and TG.  ^c^ adjusted for age, sex, BMI, TC, TG, antidiabetic medications, antihypertensive medications, physical activity, degree of education, smoking and drinking status. | | | | | | |
|  |  |  |  |  |  |  |

| **Table S7. Adjusted RR and 95% CI for CKD risk according to changes in DBP among participants of various blood glucose categories** | | | | | | |
| --- | --- | --- | --- | --- | --- | --- |
|  |  |  |  |  |  |  |
| Changes in DBP(mmHg) | n/total | Rate | Crude RR (95% CI) | Adjusted RR (95% CI) | | |
|  |  |  |  | Model 1^a^ | Model 2^b^ | Model 3^c^ |
| Total | 1355/11183 | 12.1 |  |  |  |  |
| <-5 | 438/4565 | 9.6 | 0.79(0.69-0.91) | 0.77(0.66-0.88) | 0.76(0.66-0.88) | 0.76(0.65-0.87) |
| -5 to 5 | 417/3526 | 11.8 | 1.00 (Reference) | 1.00 (Reference) | 1.00 (Reference) | 1.00 (Reference) |
| ≥5 | 500/3092 | 16.2 | 1.44(1.25-1.65) | 1.47(1.27-1.69) | 1.44(1.25-1.66) | 1.42(1.23-1.64) |
| *P*_trend_ |  | <0.001 | <0.001 | <0.001 | <0.001 | <0.001 |
| Normoglycemia | 616/4970 | 12.4 |  |  |  |  |
| <-5 | 181/1913 | 9.5 | 0.72(0.58-0.89) | 0.70(0.57-0.87) | 0.71(0.58-0.88) | 0.70(0.56-0.87) |
| -5 to 5 | 201/1590 | 12.6 | 1.00 (Reference) | 1.00 (Reference) | 1.00 (Reference) | 1.00 (Reference) |
| ≥5 | 234/1467 | 16.0 | 1.31(1.07-1.61) | 1.35(1.10-1.66) | 1.33(1.08-1.63) | 1.31(1.07-1.61) |
| *P*_trend_ |  | 0.008 | <0.001 | <0.001 | <0.001 | <0.001 |
| Prediabetes | 456/4361 | 10.5 |  |  |  |  |
| <-5 | 157/1870 | 8.4 | 0.87(0.68-1.10) | 0.85(0.67-1.09) | 0.84(0.66-1.08) | 0.85(0.66-1.09) |
| -5 to 5 | 131/1368 | 9.6 | 1.00 (Reference) | 1.00 (Reference) | 1.00 (Reference) | 1.00 (Reference) |
| ≥5 | 168/1123 | 15.0 | 1.66(1.30-2.12) | 1.69(1.32-2.15) | 1.64(1.28-2.11) | 1.65(1.28-2.12) |
| *P*_trend_ |  | <0.001 | <0.001 | <0.001 | <0.001 | <0.001 |
| Diabetes | 283/1852 | 15.3 |  |  |  |  |
| <-5 | 100/782 | 12.8 | 0.83(0.61-1.14) | 0.78(0.57-1.08) | 0.78(0.57-1.07) | 0.77(0.56-1.06) |
| -5 to 5 | 85/568 | 15.0 | 1.00 (Reference) | 1.00 (Reference) | 1.00 (Reference) | 1.00 (Reference) |
| ≥5 | 98/502 | 19.5 | 1.38(1.00-1.90) | 1.39(1.01-1.92) | 1.35(0.97-1.86) | 1.28(0.92-1.77) |
| *P*_trend_ |  | 0.050 | 0.005 | 0.001 | 0.003 | 0.006 |
| Abbreviation: DBP, diastolic blood pressure; RR, relative risk; CI, confidence interval; CKD, chronic kidney disease; BMI, body mass index; TC, total cholesterol; TG, triglyceride.  ^a^ adjusted for age and sex.  ^b^ adjusted for age, sex, BMI, TC, and TG.  ^c^ adjusted for age, sex, BMI, TC, TG, antidiabetic medications, antihypertensive medications, physical activity, degree of education, smoking and drinking status. | | | | | | |
|  |  |  |  |  |  |  |

| **Table S8. Adjusted RR and 95% CI for CKD risk according to changes in SBP among participants of various blood glucose categories without antihypertensive or antidiabetic medications at baseline** | | | | |
| --- | --- | --- | --- | --- |
|
| Changes in SBP(mmHg) | n/total | Rate | Crude RR (95% CI) | Adjusted RR (95% CI)^a^ |
|
| Total | 786/7877 | 10.0 |  |  |
| <-5 | 445/5245 | 8.5 | 0.70(0.57-0.85) | 0.69(0.56-0.84) |
| -5 to 5 | 146/1245 | 11.7 | 1.00 (Reference) | 1.00 (Reference) |
| ≥5 | 195/1387 | 14.1 | 1.23(0.98-1.55) | 1.23(0.97-1.56) |
| *P*_trend_ |  | 0.023 | <0.001 | <0.001 |
| Normoglycemia | 429/4011 | 10.7 |  |  |
| <-5 | 239/2612 | 9.2 | 0.73(0.56-0.96) | 0.73(0.55-0.96) |
| -5 to 5 | 81/669 | 12.1 | 1.00 (Reference) | 1.00 (Reference) |
| ≥5 | 109/730 | 14.9 | 1.27(0.94-1.74) | 1.32(0.96-1.82) |
| *P*_trend_ |  | 0.063 | <0.001 | <0.001 |
| Prediabetes | 294/3186 | 9.2 |  |  |
| <-5 | 169/2176 | 7.8 | 0.62(0.45-0.86) | 0.61(0.44-0.85) |
| -5 to 5 | 56/470 | 11.9 | 1.00 (Reference) | 1.00 (Reference) |
| ≥5 | 69/540 | 12.8 | 1.08(0.74-1.58) | 1.06(0.72-1.57) |
| *P*_trend_ |  | 0.477 | <0.001 | <0.001 |
| Diabetes | 63/680 | 9.3 |  |  |
| <-5 | 37/457 | 8.1 | 0.95(0.44-2.03) | 0.95(0.44-2.07) |
| -5 to 5 | 9/106 | 8.5 | 1.00 (Reference) | 1.00 (Reference) |
| ≥5 | 17/117 | 14.5 | 1.83(0.78-4.31) | 1.70(0.70-4.15) |
| *P*_trend_ |  | 0.107 | 0.103 | 0.206 |
| Abbreviation: SBP, systolic blood pressure; RR, relative risk; CI, confidence interval; CKD, chronic kidney disease; BMI, body mass index; TC, total cholesterol; TG, triglyceride.  ^a^ Adjusted for age, sex, BMI, TC, TG, physical activity, degree of education, smoking, and drinking status. | | | | |
|

| **Table S9. Adjusted RR and 95% CI for CKD risk according to changes in DBP among participants of various blood glucose categories without antihypertensive or antidiabetic medications at baseline.** | | | | |
| --- | --- | --- | --- | --- |
|
| Changes in DBP(mmHg) | n/total | Rate | Crude RR (95% CI) | Adjusted RR (95% CI)^a^ |
|
| Total | 786/7877 | 10.0 |  |  |
| <-5 | 250/3213 | 7.8 | 0.78(0.65-0.94) | 0.75(0.62-0.90) |
| -5 to 5 | 251/2574 | 9.8 | 1.00 (Reference) | 1.00 (Reference) |
| ≥5 | 285/2090 | 13.6 | 1.46(1.22-1.75) | 1.43(1.19-1.73) |
| *P*_trend_ |  | <0.001 | <0.001 | <0.001 |
| Normoglycemia | 429/4011 | 10.7 |  |  |
| <-5 | 127/1562 | 8.1 | 0.73(0.57-0.94) | 0.69(0.54-0.90) |
| -5 to 5 | 143/1320 | 10.8 | 1.00 (Reference) | 1.00 (Reference) |
| ≥5 | 159/1129 | 14.1 | 1.35(1.06-1.72) | 1.32(1.03-1.69) |
| *P*_trend_ |  | 0.017 | <0.001 | <0.001 |
| Prediabetes | 294/3186 | 9.2 |  |  |
| <-5 | 103/1364 | 7.6 | 0.89(0.66-1.19) | 0.87(0.64-1.19) |
| -5 to 5 | 87/1031 | 8.4 | 1.00 (Reference) | 1.00 (Reference) |
| ≥5 | 104/791 | 13.1 | 1.64(1.22-2.22) | 1.62(1.18-2.22) |
| *P*_trend_ |  | 0.001 | <0.001 | <0.001 |
| Diabetes | 63/680 | 9.3 |  |  |
| <-5 | 20/287 | 7.0 | 0.72(0.38-1.37) | 0.65(0.34-1.26) |
| -5 to 5 | 21/223 | 9.4 | 1.00 (Reference) | 1.00 (Reference) |
| ≥5 | 22/170 | 12.9 | 1.43(0.76-2.70) | 1.46(0.75-2.83) |
| *P*_trend_ |  | 0.302 | 0.109 | 0.068 |
| Abbreviation: DBP, diastolic blood pressure; RR, relative risk; CI, confidence interval; CKD, chronic kidney disease; BMI, body mass index; TC, total cholesterol; TG, triglyceride.  ^a^ Adjusted for age, sex, BMI, TC, TG, physical activity, degree of education, smoking, and drinking status. | | | | |
|

| **Table S10. Adjusted RR and 95% CI for CKD risk according to changes in SBP among participants of various blood glucose categories (5 categories of changes in SBP)** | | | | | | |
| --- | --- | --- | --- | --- | --- | --- |
|  |  |  |  |  |  |  |
| Changes in SBP(mmHg) | n/total | Rate | Crude RR (95% CI) | Adjusted RR (95% CI) | | |
|  |  |  |  | Model 1^a^ | Model 2^b^ | Model 3^c^ |
| Total | 1355/11183 | 12.1 |  |  |  |  |
| <-15 | 534/5055 | 10.6 | 0.77(0.66-0.91) | 0.74(0.62-0.87) | 0.73(0.62-0.87) | 0.73(0.62-0.87) |
| -15 to -5 | 239/2278 | 10.5 | 0.77(0.63-0.93) | 0.76(0.63-0.92) | 0.77(0.63-0.93) | 0.78(0.64-0.95) |
| -5 to 5 | 233/1758 | 13.3 | 1.00 (Reference) | 1.00 (Reference) | 1.00 (Reference) | 1.00 (Reference) |
| 5-15 | 174/1157 | 15.0 | 1.16(0.94-1.43) | 1.15(0.93-1.42) | 1.16(0.94-1.45) | 1.16(0.93-1.44) |
| ≥15 | 175/935 | 18.7 | 1.51(1.22-1.87) | 1.48(1.19-1.84) | 1.45(1.17-1.81) | 1.45(1.16-1.81) |
| *P*trend |  | <0.001 | <0.001 | <0.001 | <0.001 | <0.001 |
| Normoglycemia | 616/4354 | 12.4 |  |  |  |  |
| <-15 | 230/1899 | 10.8 | 0.79(0.62-1.00) | 0.74(0.58-0.95) | 0.75(0.58-0.95) | 0.75(0.59-0.96) |
| -15 to -5 | 107/931 | 10.3 | 0.75(0.56-0.99) | 0.74(0.56-0.98) | 0.75(0.56-0.99) | 0.77(0.58-1.03) |
| -5 to 5 | 112/728 | 13.3 | 1.00 (Reference) | 1.00 (Reference) | 1.00 (Reference) | 1.00 (Reference) |
| 5-15 | 83/452 | 15.5 | 1.19(0.89-1.62) | 1.20(0.88-1.64) | 1.22(0.90-1.67) | 1.25(0.92-1.71) |
| ≥15 | 84/344 | 19.6 | 1.59(1.16-2.17) | 1.55(1.14-2.13) | 1.52(1.11-2.08) | 1.50(1.09-2.06) |
| *P*trend |  | <0.001 | <0.001 | <0.001 | <0.001 | <0.001 |
| Prediabetes | 456/3905 | 10.5 |  |  |  |  |
| <-15 | 176/1869 | 8.6 | 0.67(0.50-0.88) | 0.64(0.48-0.85) | 0.64(0.48-0.85) | 0.63(0.47-0.85) |
| -15 to -5 | 86/816 | 9.5 | 0.74(0.54-1.03) | 0.74(0.53-1.03) | 0.73(0.53-1.02) | 0.74(0.53-1.04) |
| -5 to 5 | 79/558 | 12.4 | 1.00 (Reference) | 1.00 (Reference) | 1.00 (Reference) | 1.00 (Reference) |
| 5-15 | 56/383 | 12.8 | 1.03(0.72-1.49) | 1.03(0.71-1.49) | 1.03(0.71-1.50) | 1.00(0.69-1.47) |
| ≥15 | 59/279 | 17.5 | 1.49(1.04-2.16) | 1.51(1.05-2.19) | 1.47(1.01-2.10) | 1.54(1.05-2.24) |
| *P*trend |  | 0.002 | <0.001 | <0.001 | <0.001 | <0.001 |
| Diabetes | 283/1569 | 15.3 |  |  |  |  |
| <-15 | 128/753 | 14.5 | 0.97(0.66-1.41) | 0.94(0.64-1.37) | 0.95(0.65-1.39) | 0.96(0.65-1.42) |
| -15 to -5 | 46/292 | 13.6 | 0.90(0.57-1.41) | 0.92(0.58-1.45) | 0.97(0.61-1.53) | 0.99(0.62-1.58) |
| -5 to 5 | 42/239 | 14.9 | 1.00 (Reference) | 1.00 (Reference) | 1.00 (Reference) | 1.00 (Reference) |
| 5-15 | 35/148 | 19.1 | 1.35(0.82-2.20) | 1.31(0.80-2.16) | 1.36(0.82-2.25) | 1.34(0.80-2.25) |
| ≥15 | 32/137 | 18.9 | 1.33(0.80-2.20) | 1.28(0.77-2.14) | 1.33(0.80-2.23) | 1.35(0.80-2.27) |
| *P*trend |  | 0.114 | 0.294 | 0.365 | 0.332 | 0.392 |
| Abbreviation: SBP, systolic blood pressure; RR, relative risk; CI, confidence interval; CKD, chronic kidney disease; BMI, body mass index; TC, total cholesterol; TG, triglyceride.  ^a^ adjusted for age and sex.  ^b^ adjusted for age, sex, BMI, TC, and TG.  ^c^ adjusted for age, sex, BMI, TC, TG, antidiabetic medications, antihypertensive medications, physical activity, degree of education, smoking and drinking status. | | | | | | |
|  |  |  |  |  |  |  |

| **Table S11. Adjusted RR and 95% CI for CKD risk according to changes in DBP among participants of various blood glucose categories (5 categories of changes in DBP)** | | | | | | |
| --- | --- | --- | --- | --- | --- | --- |
|  |  |  |  |  |  |  |
| Changes in DBP(mmHg) | n/total | Rate | Crude RR (95% CI) | Adjusted RR (95% CI) | | |
|  |  |  |  | Model 1^a^ | Model 2^b^ | Model 3^c^ |
| Total | 1355/11183 | 12.1 |  |  |  |  |
| <-15 | 142/1615 | 8.8 | 0.72(0.59-0.88) | 0.68(0.56-0.83) | 0.68(0.55-0.83) | 0.67(0.55-0.82) |
| -15 to -5 | 296/2950 | 10.0 | 0.83(0.71-0.97) | 0.81(0.69-0.95) | 0.82(0.70-0.96) | 0.81(0.69-0.95) |
| -5 to 5 | 417/3526 | 11.8 | 1.00 (Reference) | 1.00 (Reference) | 1.00 (Reference) | 1.00 (Reference) |
| 5-15 | 354/2216 | 16.0 | 1.42(1.22-1.65) | 1.45(1.24-1.69) | 1.43(1.22-1.67) | 1.41(1.20-1.64) |
| ≥15 | 146/876 | 16.7 | 1.49(1.22-1.83) | 1.52(1.24-1.87) | 1.47(1.20-1.82) | 1.47(1.19-1.82) |
| *P*_trend_ |  | <0.001 | <0.001 | <0.001 | <0.001 | <0.001 |
| Normoglycemia | 616/4970 | 12.4 |  |  |  |  |
| <-15 | 46/627 | 7.3 | 0.55(0.39-0.77) | 0.51(0.36-0.71) | 0.51(0.37-0.72) | 0.49(0.34-0.69) |
| -15 to -5 | 135/1286 | 10.5 | 0.81(0.64-1.02) | 0.80(0.64-1.01) | 0.82(0.65-1.03) | 0.81(0.64-1.02) |
| -5 to 5 | 201/1590 | 12.6 | 1.00 (Reference) | 1.00 (Reference) | 1.00 (Reference) | 1.00 (Reference) |
| 5-15 | 167/1072 | 15.6 | 1.28(1.02-1.59) | 1.32(1.05-1.65) | 1.30(1.04-1.64) | 1.29(1.03-1.62) |
| ≥15 | 67/395 | 17.0 | 1.41(1.04-1.91) | 1.44(1.06-1.95) | 1.40(1.03-1.90) | 1.38(1.01-1.88) |
| *P*_trend_ |  | 0.002 | <0.001 | <0.001 | <0.001 | <0.001 |
| Prediabetes | 456/4361 | 10.5 |  |  |  |  |
| <-15 | 56/681 | 8.2 | 0.85(0.61-1.17) | 0.84(0.61-1.17) | 0.85(0.61-1.19) | 0.86(0.62-1.20) |
| -15 to -5 | 101/1189 | 8.5 | 0.88(0.67-1.15) | 0.86(0.65-1.13) | 0.84(0.64-1.11) | 0.84(0.63-1.11) |
| -5 to 5 | 131/1368 | 9.6 | 1.00 (Reference) | 1.00 (Reference) | 1.00 (Reference) | 1.00 (Reference) |
| 5-15 | 121/795 | 15.2 | 1.70(1.30-2.21) | 1.70(1.30-2.22) | 1.68(1.28-2.20) | 1.69(1.29-2.22) |
| ≥15 | 47/328 | 14.3 | 1.58(1.11-2.26) | 1.65(1.15-2.36) | 1.58(1.10-2.29) | 1.66(1.15-2.39) |
| *P*_trend_ |  | <0.001 | <0.001 | <0.001 | <0.001 | <0.001 |
| Diabetes | 283/1852 | 15.3 |  |  |  |  |
| <-15 | 40/307 | 13.0 | 0.85(0.57-1.28) | 0.78(0.52-1.17) | 0.78(0.51-1.17) | 0.77(0.51-1.17) |
| -15 to -5 | 60/475 | 12.6 | 0.82(0.58-1.17) | 0.79(0.55-1.13) | 0.80(0.56-1.15) | 0.80(0.56-1.15) |
| -5 to 5 | 85/568 | 15.0 | 1.00 (Reference) | 1.00 (Reference) | 1.00 (Reference) | 1.00 (Reference) |
| 5-15 | 66/349 | 18.9 | 1.33(0.93-1.89) | 1.36(0.95-1.94) | 1.32(0.92-1.89) | 1.24(0.86-1.79) |
| ≥15 | 32/153 | 20.9 | 1.50(0.96-2.36) | 1.48(0.94-2.34) | 1.50(0.95-2.37) | 1.41(0.88-2.24) |
| *P*_trend_ |  | 0.047 | 0.027 | 0.009 | 0.015 | 0.043 |
| Abbreviation: DBP, diastolic blood pressure; RR, relative risk; CI, confidence interval; CKD, chronic kidney disease; BMI, body mass index; TC, total cholesterol; TG, triglyceride.  ^a^ adjusted for age and sex.  ^b^ adjusted for age, sex, BMI, TC, and TG.  ^c^ adjusted for age, sex, BMI, TC, TG, antidiabetic medications, antihypertensive medications, physical activity, degree of education, smoking and drinking status. | | | | | | |
|  |  |  |  |  |  |  |
|  |  |  |  |  |  |  |

| **Table S12. Adjusted RR and 95% CI for rapid eGFR decline risk according to changes in SBP among participants of various blood glucose categories** | | | | |
| --- | --- | --- | --- | --- |
|
| Changes in SBP(mmHg) | n/total | Rate | Crude RR (95% CI) | Adjusted RR (95% CI)^a^ |
|
| Total | 2602/9547 | 27.3 |  |  |
| <-5 | 1590/6363 | 25.0 | 0.83(0.73-0.94) | 0.82(0.72-0.93) |
| -5 to 5 | 422/1472 | 28.7 | 1.00 (Reference) | 1.00 (Reference) |
| ≥5 | 590/1712 | 34.5 | 1.31(1.13-1.52) | 1.29(1.11-1.51) |
| *P*_trend_ |  | ＜0.001 | ＜0.001 | ＜0.001 |
| Normoglycemia | 1158/4120 | 28.1 |  |  |
| <-5 | 696/2685 | 25.9 | 0.87(0.72-1.05) | 0.85(0.71-1.03) |
| -5 to 5 | 196/682 | 28.7 | 1.00 (Reference) | 1.00 (Reference) |
| ≥5 | 266/753 | 35.3 | 1.35(1.08-1.69) | 1.37(1.09-1.71) |
| *P*_trend_ |  | 0.003 | ＜0.001 | ＜0.001 |
| Prediabetes | 953/3811 | 25.0 |  |  |
| <-5 | 587/2605 | 22.5 | 0.76(0.62-0.93) | 0.75(0.61-0.92) |
| -5 to 5 | 153/551 | 27.8 | 1.00 (Reference) | 1.00 (Reference) |
| ≥5 | 213/655 | 32.5 | 1.25(0.98-1.61) | 1.25(0.97-1.60) |
| *P*_trend_ |  | 0.024 | ＜0.001 | ＜0.001 |
| Diabetes | 491/1616 | 30.4 |  |  |
| <-5 | 307/1073 | 28.6 | 0.91(0.67-1.24) | 0.92(0.68-1.25) |
| -5 to 5 | 73/239 | 30.5 | 1.00 (Reference) | 1.00 (Reference) |
| ≥5 | 111/304 | 36.5 | 1.31(0.91-1.88) | 1.30(0.90-1.87) |
| *P*_trend_ |  | 0.088 | 0.031 | 0.044 |
| Abbreviation: SBP, systolic blood pressure; RR, relative risk; CI, confidence interval; eGFR, estimated glomerular filtration rate; BMI, body mass index; TC, total cholesterol; TG, triglyceride.  ^a^ Adjusted for age, sex, BMI, TC, TG, antidiabetic medications, antihypertensive medications, physical activity, degree of education, smoking and drinking status. | | | | |
|

| **Table S13. Adjusted RR and 95% CI for rapid eGFR decline risk according to changes in DBP among participants of various blood glucose categories** | | | | |
| --- | --- | --- | --- | --- |
|
| Changes in DBP(mmHg) | n/total | Rate | Crude RR (95% CI) | Adjusted RR (95% CI)^a^ |
|
| Total | 2602/9547 | 27.3 |  |  |
| <-5 | 948/4066 | 23.3 | 0.83(0.75-0.93) | 0.82(0.73-0.91) |
| -5 to 5 | 809/3010 | 26.8 | 1.00 (Reference) | 1.00 (Reference) |
| ≥5 | 845/2463 | 34.3 | 1.43(1.27-1.60) | 1.41(1.25-1.58) |
| *P*_trend_ |  | ＜0.001 | ＜0.001 | ＜0.001 |
| Normoglycemia | 1158/4120 | 28.1 |  |  |
| <-5 | 390/1656 | 23.6 | 0.80(0.68-0.94) | 0.78(0.66-0.93) |
| -5 to 5 | 370/1331 | 27.8 | 1.00 (Reference) | 1.00 (Reference) |
| ≥5 | 398/1133 | 35.1 | 1.41(1.19-1.67) | 1.41(1.19-1.68) |
| *P*_trend_ |  | ＜0.001 | ＜0.001 | ＜0.001 |
| Prediabetes | 953/3811 | 25.0 |  |  |
| <-5 | 364/1704 | 21.4 | 0.83(0.70-1.00) | 0.83(0.70-0.99) |
| -5 to 5 | 292/1189 | 24.6 | 1.00 (Reference) | 1.00 (Reference) |
| ≥5 | 297/918 | 32.4 | 1.47(1.21-1.78) | 1.46(1.20-1.77) |
| *P*_trend_ |  | ＜0.001 | ＜0.001 | ＜0.001 |
| Diabetes | 491/1616 | 30.4 |  |  |
| <-5 | 194/706 | 27.5 | 0.91(0.70-1.17) | 0.91(0.70-1.17) |
| -5 to 5 | 147/498 | 29.5 | 1.00 (Reference) | 1.00 (Reference) |
| ≥5 | 150/421 | 36.4 | 1.37(1.04-1.81) | 1.33(1.01-1.76) |
| *P*_trend_ |  | 0.035 | 0.007 | 0.014 |
| Abbreviation: DBP, diastolic blood pressure; RR, relative risk; CI, confidence interval; eGFR, estimated glomerular filtration rate; BMI, body mass index; TC, total cholesterol; TG, triglyceride.  ^a^ Adjusted for age, sex, BMI, TC, TG, antidiabetic medications, antihypertensive medications, physical activity, degree of education, smoking and drinking status. | | | | |
|

| **Table S14. Subgroup analysis of associations between BP categories and risk of incident CKD among participants of various blood glucose categories^a^** | | | | | | |
| --- | --- | --- | --- | --- | --- | --- |
|  |  |  |  |  |  |  |
|  |  | **SBP/DBP Categories (mmHg)** | | | *P* _trend_ | *P_Interaction_* |
|  |  | < 130/80 | 130-139/80-89 | ≥140/90 |  |  |
| **Total** |  |  |  |  |  | 0.200 |
|  | Normoglycemia | 1.00 (Reference) | 1.08(0.92-1.27) | 1.56(1.32-1.83) | <0.001 |  |
|  | Prediabetes | 1.00 (Reference) | 1.19(0.98-1.45) | 1.59(1.32-1.92) | <0.001 |  |
|  | Diabetes | 1.00 (Reference) | 1.51(1.14-2.01) | 1.89(1.44-2.46) | <0.001 |  |
| **Sex** | **Men** |  |  |  |  | 0.974 |
|  | Normoglycemia | 1.00 (Reference) | 1.17(0.88-1.56) | 1.64(1.24-2.16) | 0.001 |  |
|  | Prediabetes | 1.00 (Reference) | 1.25(0.92-1.71) | 1.74(1.31-2.31) | <0.001 |  |
|  | Diabetes | 1.00 (Reference) | 1.32(0.86-2.03) | 1.59(1.06-2.38) | 0.077 |  |
|  | **Women** |  |  |  |  | 0.128 |
|  | Normoglycemia | 1.00 (Reference) | 1.04(0.85-1.28) | 1.55(1.26-1.90) | <0.001 |  |
|  | Prediabetes | 1.00 (Reference) | 1.21(0.93-1.57) | 1.56(1.21-2.00) | 0.002 |  |
|  | Diabetes | 1.00 (Reference) | 1.69(1.15-2.49) | 2.09(1.46-3.00) | <0.001 |  |
| **Age** | **Age < 60 years** |  |  |  |  | 0.255 |
|  | Normoglycemia | 1.00 (Reference) | 1.00(0.77-1.32) | 1.54(1.16-2.04) | 0.003 |  |
|  | Prediabetes | 1.00 (Reference) | 1.13(0.78-1.64) | 1.47(1.02-2.11) | 0.102 |  |
|  | Diabetes | 1.00 (Reference) | 1.35(0.70-2.62) | 2.09(1.13-3.84) | 0.047 |  |
|  | **Age ≥ 60 years** |  |  |  |  | 0.405 |
|  | Normoglycemia | 1.00 (Reference) | 1.13(0.92-1.40) | 1.60(1.30-1.96) | <0.001 |  |
|  | Prediabetes | 1.00 (Reference) | 1.24(0.98-1.57) | 1.67(1.34-2.09) | <0.001 |  |
|  | Diabetes | 1.00 (Reference) | 1.52(1.10-2.10) | 1.79(1.33-2.42) | 0.001 |  |
| **BMI** | **BMI < 24 kg/m^2^** |  |  |  |  | 0.440 |
|  | Normoglycemia | 1.00 (Reference) | 1.13(0.90-1.42) | 1.66(1.32-2.07) | <0.001 |  |
|  | Prediabetes | 1.00 (Reference) | 1.41(1.03-1.92) | 1.70(1.26-2.30) | 0.002 |  |
|  | Diabetes | 1.00 (Reference) | 2.37(1.44-3.91) | 2.26(1.40-3.65) | 0.001 |  |
|  | **BMI ≥ 24 kg/m^2^** |  |  |  |  | 0.367 |
|  | Normoglycemia | 1.00 (Reference) | 1.07(0.85-1.36) | 1.56(1.24-1.96) | <0.001 |  |
|  | Prediabetes | 1.00 (Reference) | 1.11(0.86-1.42) | 1.55(1.23-1.96) | <0.001 |  |
|  | Diabetes | 1.00 (Reference) | 1.28(0.91-1.81) | 1.83(1.33-2.51) | <0.001 |  |
| **Smoking status** | **Never-smokers** |  |  |  |  | 0.465 |
|  | Normoglycemia | 1.00 (Reference) | 1.07(0.89-1.29) | 1.48(1.22-1.78) | <0.001 |  |
|  | Prediabetes | 1.00 (Reference) | 1.20(0.95-1.51) | 1.66(1.34-2.05) | <0.001 |  |
|  | Diabetes | 1.00 (Reference) | 1.36(0.98-1.89) | 1.66(1.22-2.25) | 0.005 |  |
|  | **Ever-smokers** |  |  |  |  | 0.155 |
|  | Normoglycemia | 1.00 (Reference) | 1.16(0.84-1.60) | 1.89(1.38-2.57) | <0.001 |  |
|  | Prediabetes | 1.00 (Reference) | 1.34(0.83-1.94) | 1.75(1.24-2.46) | 0.006 |  |
|  | Diabetes | 1.00 (Reference) | 2.06(1.17-3.64) | 2.79(1.66-4.71) | 0.001 |  |
| **Drinking status** | **Never-drinkers** |  |  |  |  | 0.256 |
|  | Normoglycemia | 1.00 (Reference) | 1.02(0.85-1.23) | 1.46(1.22-1.76) | <0.001 |  |
|  | Prediabetes | 1.00 (Reference) | 1.17(0.93-1.46) | 1.57(1.27-1.95) | <0.001 |  |
|  | Diabetes | 1.00 (Reference) | 1.53(1.11-2.11) | 1.86(1.38-2.51) | <0.001 |  |
|  | **Eever-drinkers** |  |  |  |  | 0.529 |
|  | Normoglycemia | 1.00 (Reference) | 1.34(0.94-1.92) | 1.95(1.39-2.74) | <0.001 |  |
|  | Prediabetes | 1.00 (Reference) | 1.44(0.97-2.14) | 1.92(1.34-2.75) | 0.002 |  |
|  | Diabetes | 1.00 (Reference) | 1.51(0.83-2.78) | 2.12(1.23-3.66) | 0.024 |  |
| **Baseline renal function** | **eGFR < 90mL/min/1.73m^2^** |  |  |  |  | 0.275 |
|  | Normoglycemia | 1.00 (Reference) | 1.14(0.93-1.39) | 1.63(1.34-1.98) | <0.001 |  |
|  | Prediabetes | 1.00 (Reference) | 1.11(0.88-1.39) | 1.53(1.23-1.90) | <0.001 |  |
|  | Diabetes | 1.00 (Reference) | 1.55(1.11-2.18) | 1.97(1.44-2.69) | <0.001 |  |
|  | **eGFR ≥ 90 mL/min/1.73m^2^** |  |  |  |  | 0.435 |
|  | Normoglycemia | 1.00 (Reference) | 0.97(0.72-1.29) | 1.40(1.04-1.88) | 0.031 |  |
|  | Prediabetes | 1.00 (Reference) | 1.46(0.98-2.17) | 1.85(1.27-2.68) | 0.005 |  |
|  | Diabetes | 1.00 (Reference) | 1.30(0.75-2.26) | 1.60(0.95-2.68) | 0.210 |  |
| Abbreviation: BP, blood pressure; RR, relative risk; CI, confidence interval; CKD, chronic kidney disease; BMI, body mass index; eGFR, estimated glomerular filtration rate; TC, total cholesterol; TG, triglyceride.  ^a^ Adjusted for age, sex, BMI, TC, TG, antidiabetic medications, antihypertensive medications, physical activity, degree of education, smoking, and drinking status. | | | | | | |
|  |  |  |  |  |  |  |

| **Table S15. Subgroup analysis of associations between changes in SBP and risk of incident CKD among participants of various blood glucose categories^a^** | | | | | | |
| --- | --- | --- | --- | --- | --- | --- |
|  |  |  |  |  |  |  |
|  |  | **Changes in SBP (mmHg)** | | | *P* _trend_ | *P_Interaction_* |
|  |  | < -5 | -5 to 5 | ≥5 |  |  |
| **Total** |  |  |  |  |  | 0.680 |
|  | Normoglycemia | 0.76(0.60-0.96) | 1.00 (Reference) | 1.34(1.03-1.75) | <0.001 |  |
|  | Prediabetes | 0.66(0.51-0.87) | 1.00 (Reference) | 1.22(0.89-1.68) | <0.001 |  |
|  | Diabetes | 0.97(0.66-1.41) | 1.00 (Reference) | 1.33(0.86-2.06) | 0.130 |  |
| **Sex** | **Men** |  |  |  |  | 0.274 |
|  | Normoglycemia | 0.86(0.20-1.23) | 1.00 (Reference) | 1.18(0.77-1.80) | 0.161 |  |
|  | Prediabetes | 0.90(0.58-1.38) | 1.00 (Reference) | 1.31(0.79-2.16) | 0.117 |  |
|  | Diabetes | 1.40(0.79-2.48) | 1.00 (Reference) | 1.93(1.00-3.74) | 0.140 |  |
|  | **Women** |  |  |  |  | 0.148 |
|  | Normoglycemia | 0.69(0.50-0.95) | 1.00 (Reference) | 1.49(1.06-2.10) | <0.001 |  |
|  | Prediabetes | 0.52(0.36-0.75) | 1.00 (Reference) | 1.20(0.79-1.82) | <0.001 |  |
|  | Diabetes | 0.68(0.41-1.13) | 1.00 (Reference) | 0.95(0.53-1.72) | 0.169 |  |
| **Age** | **Age < 60 years** |  |  |  |  | 0.195 |
|  | Normoglycemia | 0.60(0.40-0.90) | 1.00 (Reference) | 1.16(0.74-1.82) | 0.001 |  |
|  | Prediabetes | 0.54(0.31-0.94) | 1.00 (Reference) | 1.75(0.97-3.14) | <0.001 |  |
|  | Diabetes | 0.86(0.35-2.08) | 1.00 (Reference) | 1.75(0.67-4.60) | 0.125 |  |
|  | **Age ≥ 60 years** |  |  |  |  | 0.248 |
|  | Normoglycemia | 0.85(0.63-1.13) | 1.00 (Reference) | 1.50(1.08-2.10) | <0.001 |  |
|  | Prediabetes | 0.69(0.50-0.96) | 1.00 (Reference) | 1.05(0.72-1.54) | 0.005 |  |
|  | Diabetes | 1.00(0.66-1.53) | 1.00 (Reference) | 1.24(0.76-2.04) | 0.503 |  |
| **BMI** | **BMI < 24 kg/m**^2^ |  |  |  |  | 0.664 |
|  | Normoglycemia | 0.87(0.62-1.21) | 1.00 (Reference) | 1.30(0.88-1.94) | 0.035 |  |
|  | Prediabetes | 0.51(0.33-0.78) | 1.00 (Reference) | 0.67(0.39-1.14) | 0.007 |  |
|  | Diabetes | 1.60(0.76-3.41) | 1.00 (Reference) | 1.81(0.77-4.29) | 0.379 |  |
|  | **BMI ≥ 24 kg/m**^2^ |  |  |  |  | 0.635 |
|  | Normoglycemia | 0.66(0.48-0.92) | 1.00 (Reference) | 1.34(0.94-1.92) | <0.001 |  |
|  | Prediabetes | 0.76(0.53-1.10) | 1.00 (Reference) | 1.69(1.13-2.53) | <0.001 |  |
|  | Diabetes | 0.80(0.51-1.24) | 1.00 (Reference) | 1.20(0.72-2.01) | 0.097 |  |
| **Smoking status** | **Never-smokers** |  |  |  |  | 0.503 |
|  | Normoglycemia | 0.72(0.55-0.95) | 1.00 (Reference) | 1.40(1.02-1.92) | <0.001 |  |
|  | Prediabetes | 0.63(0.45-0.87) | 1.00 (Reference) | 1.37(0.94-1.99) | <0.001 |  |
|  | Diabetes | 0.91(0.57-1.43) | 1.00 (Reference) | 1.20(0.71-2.03) | 0.350 |  |
|  | **Ever-smokers** |  |  |  |  | 0.712 |
|  | Normoglycemia | 0.83(0.53-1.29) | 1.00 (Reference) | 1.22(0.74-2.03) | 0.142 |  |
|  | Prediabetes | 0.76(0.45-1.27) | 1.00 (Reference) | 0.93(0.51-1.73) | 0.464 |  |
|  | Diabetes | 1.05(0.54-2.06) | 1.00 (Reference) | 1.62(0.73-3.58) | 0.330 |  |
| **Drinking status** | **Never-drinkers** |  |  |  |  | 0.750 |
|  | Normoglycemia | 0.77(0.59-1.01) | 1.00 (Reference) | 1.34(0.98-1.82) | <0.001 |  |
|  | Prediabetes | 0.61(0.45-0.84) | 1.00 (Reference) | 1.14(0.79-1.64) | <0.001 |  |
|  | Diabetes | 0.97(0.62-1.50) | 1.00 (Reference) | 1.31(0.79-2.18) | 0.273 |  |
|  | **Eever-drinkers** |  |  |  |  | 0.885 |
|  | Normoglycemia | 0.73(0.45-1.18) | 1.00 (Reference) | 1.42(0.84-2.42) | 0.005 |  |
|  | Prediabetes | 0.88(0.49-1.59) | 1.00 (Reference) | 1.64(0.84-3.17) | 0.026 |  |
|  | Diabetes | 0.89(0.43-1.87) | 1.00 (Reference) | 1.27(0.53-3.05) | 0.568 |  |
| **Baseline renal function** | **eGFR < 90mL/min/1.73m**^2^ |  |  |  |  | 0.991 |
|  | Normoglycemia | 0.71(0.53-0.94) | 1.00 (Reference) | 1.12(0.80-1.56) | 0.001 |  |
|  | Prediabetes | 0.60(0.44-0.83) | 1.00 (Reference) | 1.02(0.70-1.49) | <0.001 |  |
|  | Diabetes | 0.92(0.59-1.44) | 1.00 (Reference) | 1.14(0.68-1.93) | 0.554 |  |
|  | **eGFR ≥ 90 mL/min/1.73m**^2^ |  |  |  |  | 0.863 |
|  | Normoglycemia | 0.82(0.54-1.26) | 1.00 (Reference) | 1.82(1.15-2.88) | <0.001 |  |
|  | Prediabetes | 0.86(0.48-1.53) | 1.00 (Reference) | 2.12(1.12-3.98) | <0.001 |  |
|  | Diabetes | 1.06(0.51-2.18) | 1.00 (Reference) | 1.79(0.79-4.06) | 0.171 |  |
| Abbreviation: SBP, systolic blood pressure; RR, relative risk; CI, confidence interval; CKD, chronic kidney disease; BMI, body mass index; eGFR, estimated glomerular filtration rate; TC, total cholesterol; TG, triglyceride.  ^a^ Adjusted for age, sex, BMI, TC, TG, antidiabetic medications, antihypertensive medications, physical activity, degree of education, smoking, and drinking status. | | | | | | |
|  |  |  |  |  |  |  |

| **Table S16. Subgroup analysis of associations between changes in DBP and risk of incident CKD among participants of various blood glucose categories^a^** | | | | | | |
| --- | --- | --- | --- | --- | --- | --- |
|  |  |  |  |  |  |  |
|  |  | **Changes in DBP (mmHg)** | | | *P* _trend_ | *P_Interaction_* |
|  |  | < -5 | -5 to 5 | ≥5 |  |  |
| **Total** | **Total** |  |  |  |  | 0.837 |
|  | Normoglycemia | 0.70(0.56-0.87) | 1.00 (Reference) | 1.31(1.07-1.61) | <0.001 |  |
|  | Prediabetes | 0.85(0.66-1.09) | 1.00 (Reference) | 1.65(1.28-2.12) | <0.001 |  |
|  | Diabetes | 0.77(0.56-1.06) | 1.00 (Reference) | 1.28(0.92-1.77) | 0.009 |  |
| **Sex** | **Men** |  |  |  |  | 0.837 |
|  | Normoglycemia | 0.89(0.64-1.24) | 1.00 (Reference) | 1.44(1.03-2.01) | 0.011 |  |
|  | Prediabetes | 0.80(0.56-1.14) | 1.00 (Reference) | 1.19(0.81-1.75) | 0.014 |  |
|  | Diabetes | 1.44(0.89-2.32) | 1.00 (Reference) | 2.16(1.28-3.64) | 0.099 |  |
|  | **Women** |  |  |  |  | 0.402 |
|  | Normoglycemia | 0.59(0.44-0.79) | 1.00 (Reference) | 1.24(0.95-1.62) | <0.001 |  |
|  | Prediabetes | 0.89(0.62-1.27) | 1.00 (Reference) | 2.13(1.51-2.99) | <0.001 |  |
|  | Diabetes | 0.46(0.29-0.72) | 1.00 (Reference) | 0.89(0.57-1.37) | 0.002 |  |
| **Age** | **Age < 60 years** |  |  |  |  | 0.842 |
|  | Normoglycemia | 0.54(0.36-0.81) | 1.00 (Reference) | 1.10(0.77-1.57) | 0.001 |  |
|  | Prediabetes | 0.76(0.43-1.32) | 1.00 (Reference) | 2.24(1.37-3.65) | <0.001 |  |
|  | Diabetes | 0.29(0.13-0.63) | 1.00 (Reference) | 0.56(0.28-1.15) | 0.008 |  |
|  | **Age ≥ 60 years** |  |  |  |  | 0.773 |
|  | Normoglycemia | 0.79(0.60-1.02) | 1.00 (Reference) | 1.45(1.12-1.88) | <0.001 |  |
|  | Prediabetes | 0.86(0.65-1.15) | 1.00 (Reference) | 1.47(1.09-1.98) | 0.001 |  |
|  | Diabetes | 0.99(0.69-1.42) | 1.00 (Reference) | 1.58(1.08-2.31) | 0.015 |  |
| **BMI** | **BMI < 24 kg/m^2^** |  |  |  |  | 0.195 |
|  | Normoglycemia | 0.66(0.49-0.89) | 1.00 (Reference) | 1.05(0.77-1.42) | 0.005 |  |
|  | Prediabetes | 0.86(0.58-1.29) | 1.00 (Reference) | 1.77(1.16-2.69) | 0.001 |  |
|  | Diabetes | 0.66(0.37-1.18) | 1.00 (Reference) | 1.37(0.75-2.49) | 0.051 |  |
|  | **BMI ≥ 24 kg/m^2^** |  |  |  |  | 0.283 |
|  | Normoglycemia | 0.74(0.54-1.02) | 1.00 (Reference) | 1.59(1.19-2.12） | <0.001 |  |
|  | Prediabetes | 0.82(0.60-1.13) | 1.00 (Reference) | 1.56(1.14-2.14) | <0.001 |  |
|  | Diabetes | 0.84(0.57-1.23) | 1.00 (Reference) | 1.26(0.85-1.88) | 0.098 |  |
| **Smoking status** | **Never-smokers** |  |  |  |  | 0.601 |
|  | Normoglycemia | 0.70(0.54-0.91) | 1.00 (Reference) | 1.26(0.99-1.62) | <0.001 |  |
|  | Prediabetes | 0.80(0.59-1.08) | 1.00 (Reference) | 1.69(1.26-2.27) | <0.001 |  |
|  | Diabetes | 0.61(0.42-0.89) | 1.00 (Reference) | 0.98(0.67-1.45) | 0.016 |  |
|  | **Ever-smokers** |  |  |  |  | 0.208 |
|  | Normoglycemia | 0.72(0.48-1.08) | 1.00 (Reference) | 1.44(0.97-2.12) | 0.003 |  |
|  | Prediabetes | 0.96(0.62-1.50) | 1.00 (Reference) | 1.53(0.94-2.48) | 0.095 |  |
|  | Diabetes | 1.37(0.75-2.52) | 1.00 (Reference) | 2.45(1.27-4.71) | 0.020 |  |
| **Drinking status** | **Never-drinkers** |  |  |  |  | 0.589 |
|  | Normoglycemia | 0.70(0.55-1.90) | 1.00 (Reference) | 1.18(0.93-1.50) | <0.001 |  |
|  | Prediabetes | 0.84(0.63-1.13) | 1.00 (Reference) | 1.71(1.28-2.29) | <0.001 |  |
|  | Diabetes | 0.66(0.46-0.96) | 1.00 (Reference) | 1.15(0.79-1.68) | 0.010 |  |
|  | **Eever-drinkers** |  |  |  |  | 0.545 |
|  | Normoglycemia | 0.72(0.46-1.12) | 1.00 (Reference) | 1.82(1.20-2.74) | <0.001 |  |
|  | Prediabetes | 0.85(0.53-1.34) | 1.00 (Reference) | 1.43(0.87-2.35) | 0.076 |  |
|  | Diabetes | 1.23(0.64-2.36) | 1.00 (Reference) | 1.75(0.87-3.52) | 0.266 |  |
| **Baseline renal function** | **eGFR < 90mL/min/1.73m^2^** |  |  |  |  | 0.544 |
|  | Normoglycemia | 0.67(0.51-0.87) | 1.00 (Reference) | 1.21(0.93-1.57) | <0.001 |  |
|  | Prediabetes | 0.85(0.64-1.13) | 1.00 (Reference) | 1.57(1.17-2.12) | <0.001 |  |
|  | Diabetes | 0.80(0.55-1.17) | 1.00 (Reference) | 1.25(0.84-1.87) | 0.066 |  |
|  | **eGFR ≥ 90 mL/min/1.73m^2^** |  |  |  |  | 0.811 |
|  | Normoglycemia | 0.75(0.52-1.10) | 1.00 (Reference) | 1.46(1.03-2.07) | 0.001 |  |
|  | Prediabetes | 0.78(0.47-1.29) | 1.00 (Reference) | 1.78(1.11-2.85) | 0.002 |  |
|  | Diabetes | 0.72(0.39-1.32) | 1.00 (Reference) | 1.31(0.73-2.37) | 0.129 |  |
| Abbreviation: DBP, diastolic blood pressure; RR, relative risk; CI, confidence interval; CKD, chronic kidney disease; BMI, body mass index; eGFR, estimated glomerular filtration rate; TC, total cholesterol; TG, triglyceride.  ^a^ Adjusted for age, sex, BMI, TC, TG, antidiabetic medications, antihypertensive medications, physical activity, degree of education, smoking, and drinking status. | | | | | | |
